# Comparative Metabolite and Gene Expression Analyses in Combination With Gene Characterization Revealed the Patterns of Flavonoid Accumulation During *Cistus creticus* subsp. *creticus* Fruit Development

**DOI:** 10.3389/fpls.2021.619634

**Published:** 2021-03-26

**Authors:** Neda Aničić, Efstathia Patelou, Antigoni Papanikolaou, Anthi Kanioura, Camilla Valdesturli, Panagiotis Arapitsas, Marijana Skorić, Milan Dragićević, Uroš Gašić, Athanasios Koukounaras, Stefanos Kostas, Eirini Sarrou, Stefan Martens, Danijela Mišić, Angelos Kanellis

**Affiliations:** ^1^Group of Biotechnology of Pharmaceutical Plants, Laboratory of Pharmacognosy, Department of Pharmaceutical Sciences, Aristotle University of Thessaloniki, Thessaloniki, Greece; ^2^Department of Plant Physiology, Institute for Biological Research “Siniša Stanković”-National Institute of Republic of Serbia, University of Belgrade, Belgrade, Serbia; ^3^Research and Innovation Centre, Fondazione Edmund Mach, San Michele all’Adige, Italy; ^4^Department of Horticulture, School of Agriculture, Aristotle University of Thessaloniki, Thessaloniki, Greece; ^5^Institute of Plant Breeding and Genetic Resources, Hellenic Agricultural Organization - DEMETER, Thessaloniki, Greece

**Keywords:** *Cistus creticus*, flavonoids, fruit, gene expression, flavonoid hydroxylase

## Abstract

*Cistus creticus* L. subsp. *creticus* (rockrose) is a shrub widespread in Greece and the Mediterranean basin and has been used in traditional medicine as herb tea for colds, for healing and digestive hitches, for the treatment of maladies, as perfumes, and for other purposes. Compounds from its flavonoid fraction have recently drawn attention due to antiviral action against influenza virus and HIV. Although several bioactive metabolites belonging to this group have been chemically characterized in the leaves, the genes involved in their biosynthesis in *Cistus* remain largely unknown. Flavonoid metabolism during *C. creticus* fruit development was studied by adopting comparative metabolomic and transcriptomic approaches. The present study highlights the fruit of *C. creticus* subsp. c*reticus* as a rich source of flavonols, flavan-3-ols, and proanthocyanidins, all of which displayed a decreasing trend during fruit development. The majority of proanthocyanidins recorded in *Cistus* fruit are B-type procyanidins and prodelphinidins, while gallocatechin and catechin are the dominant flavan-3-ols. The expression patterns of biosynthetic genes and transcription factors were analyzed in flowers and throughout three fruit development stages. Flavonoid biosynthetic genes were developmentally regulated, showing a decrease in transcript levels during fruit maturation. A high degree of positive correlations between the content of targeted metabolites and the expression of biosynthetic genes indicated the transcriptional regulation of flavonoid biosynthesis during *C. creticus* fruit development. This is further supported by the high degree of significant positive correlations between the expression of biosynthetic genes and transcription factors. The results suggest that leucoanthocyanidin reductase predominates the biosynthetic pathway in the control of flavan-3-ol formation, which results in catechin and gallocatechin as two of the major building blocks for *Cistus* proanthocyanidins. Additionally, there is a decline in ethylene production rates during non-climacteric *Cistus* fruit maturation, which coincides with the downregulation of the majority of flavonoid- and ethylene-related biosynthetic genes and corresponding transcription factors as well as with the decline in flavonoid content. Finally, functional characterization of a *Cistus* flavonoid hydroxylase (F3′5′H) was performed for the first time.

## Introduction

**FIGURE 1:**
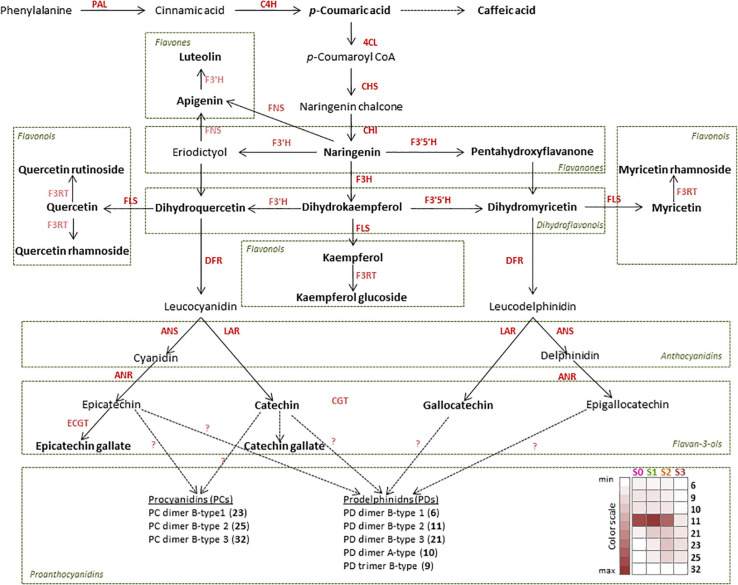
Proposed flavonoid biosynthetic pathway in *Cistus creticus* subsp. *creticus* fruit. Compounds in boldface letters are detected in flowers and in fruit of different developmental stages. Enzymes in boldface letters are those for which the gene expression analysis was performed: phenylalanine ammonia-lyase (PAL), cinnamate 4-hydroxylase (C4H), 4-coumarate:coenzyme A ligase (4CL), chalcone synthase (CHS), chalcone isomerase (CHI), flavanone-3β-hydroxylase (F3H), dihydroflavonol-4-reductase (DFR), flavonoid-3′-hydroxylase (F3′H), flavonoid 3′,5′-hydroxylase (F3′5′H), flavonol synthase (FLS), leucoanthocyanidin reductase (LAR), anthocyanidin synthase (ANS), and anthocyanidin reductase (ANR). Heat map based on the peak areas presents the amounts of proanthocyanidins (PAs) in *Cistus* flowers (S0) and fruits of three developmental stages (S1–S3). The values are represented by the intensity of the red color as indicated on the color scale.

Flavonoids are plant polyphenolic compounds synthesized through the phenylpropanoid pathway. Based on their structure, they can be classified into the major groups of flavanones, flavones, isoflavones, dihydroflavonols, flavonols, leucoanthocyanidins (flavan-3,4-diols), anthocyanidins, flavan-3-ols (F3Os), the polymeric proanthocyanidins (PAs), and anthocyanins (Marchiosi et al., 2020). An initial “core phenylpropanoid pathway” involves phenylalanine ammonia-lyase (PAL), cinnamate 4-hydroxylase (C4H), and 4-coumarate-CoA ligase (4CL), leading to 4-coumaroyl-CoA, which is the general precursor of flavonoid metabolism (Figure 1). The flavonoid pathway further proceeds *via* chalcone synthase (CHS) and chalcone isomerase (CHI) to synthesize the flavanone naringenin, which is considered the first flavonoid and the branching point of the flavonoid pathway. Naringenin can be further converted into dihydroflavonols, leading to flavonols, leucoanthocyanidins, anthocyanidins, F3Os, and PAs, respectively (Tohge et al., 2017). In another branch, naringenin and other flavanones can be converted into flavones by flavone synthases (FNSI and FNSII) (Martens and Mithöfer, 2005). The biosynthetic route leading to flavonols includes flavanone-3β-hydroxylase (F3H syn. FHT) and flavonol synthase (FLS) proteins. The number of hydroxyl groups on the B-ring (Supplementary Figure 1) is determined by the presence and activity of flavonoid 3′-hydroxylase (F3′H) and flavonoid 3′,5′-hydroxylases (F3′5′H), catalyzing the hydroxylation of the 3′ position, and the 3′ and 5′ positions, respectively (Tanaka and Brugliera, 2013). These enzymes are believed to predominantly hydroxylate dihydroflavonol substrates (Ishiguro et al., 2012), which are upstream intermediates in the flavonoid biosynthesis pathway two steps before F3Os. Two biosynthetic branches of a different stereo-configuration preference control the formation of F3Os. Leucoanthocyanidin reductase (LAR) catalyzes, in a one-step reaction, the formation of one stereotype of F3Os, 2R,3S-*trans*-flavan-3-ols [e.g., (+)-catechin], from leucoanthocyanidins. In a second branch, the anthocyanidin reductase (ANR) converts anthocyanidins, which are formed from leucoanthocyanidins in a reaction catalyzed by anthocyanidin synthase (ANS syn. leucoanthocyanidin dioxygenase, LDOX) into the other stereotype of F3Os, 2R,3R-*cis*-flavan-3-ols [e.g., (−)-epicatechin]. PAs (or condensed tannins) are polymeric flavonoids built from F3O units. The exact mechanism of PA polymerization and extension and the factors affecting the PA chain subunit composition are not fully elucidated; however, some recent studies proposed a significant role of LAR, ANS/LDOX, and ANR in this process (Liu C. et al., 2016; Jun et al., 2018). The enzymes responsible for galloylation of F3Os, known from *Camellia sinensis*, are epicatechin:1-*O*-galloyl-β*-*D-glucose *O*-galloyltransferase (ECGT) and UDP-glucose:galloyl-1-*O*-β-D-glucosyltransferase (UGGT), and they belong to the family of serine carboxypeptidase-like acyltransferases (Liu et al., 2012).

As the F3O and PA biosynthetic route is a branch of the flavonoid pathway, it has to be co-ordinately regulated with other flavonoid branches in different tissues and organs and in response to various developmental and environmental cues. Biosynthetic genes are turned on and off by specific transcription factors (TFs), which can simultaneously control several genes, or a single step within the pathway. Among plant TFs, the MYB factor proteins (V-myb myeloblastosis viral oncogene homolog) are most important within the flavonoid pathway (Martin et al., 2001). Following MYB factor binding to specific DNA regulatory elements in the promoter regions of target genes, transcriptional activation is initiated. Unlike many others, the PA- and anthocyanin-specific MYBs also need to partner with basic helix–loop–helix (bHLH) and WD-40 repeat proteins, forming the so-called MBW complex to promote transcription (Feller et al., 2011; Montefiori et al., 2015).

Most of the species belonging to the genus *Cistus* L. (Cistaceae) are spread in the Mediterranean area (Ferrer-Gallego et al., 2013) and are traditionally used as remedy for various skin diseases, as anti-inflammatory agents, and as antidiarrheics (Attaguile et al., 2000). Strong antimicrobial (Hutschenreuther et al., 2010) and cytotoxic activities (Chinou et al., 1994; Skorić et al., 2012) are usually ascribed to labdane diterpenes, which are the major components of “Oleoresin Labdanum” produced by the majority of *Cistus* species (Demetzos et al., 1997). Species from this genus are also rich sources of flavonoid and phenolic acid derivatives (Vogt et al., 1987; Demetzos et al., 1989; Pomponio et al., 2003; Barrajón-Catalán et al., 2011; Tomás-Menor et al., 2013; Papaefthimiou et al., 2014; Maggi et al., 2016). Recently, it was shown that fractions enriched in phenolic compounds from the leaves of *C. creticus* L. subsp. *creticus* [syn. *Cistus incanus* subsp. *creticus* (L.) Heywood, The Euro + Med PlantBase] exhibited potent activity against influenza virus (Droebner et al., 2007; Ehrhardt et al., 2007, 2013; Ludwig, 2011; Khoufache et al., 2013) and HIV (Rebensburg et al., 2016).

The aim of the present work was to analyze the polyphenolic composition of *C. creticus* subsp. *creticus* flowers and fruit, which, to our knowledge, have not been phytochemically characterized before. Species belonging to the genus *Cistus* primarily develop five-valve fruits (Demoly and Montserrat, 1993), with colors varying from green in juvenile to dark red in ripe fruit. Similarly, as in other fruit species, *Cistus* fruit development from flower to ripe stage undergoes metabolic alterations regulated by both developmental and hormonal factors. The accumulation of major groups of flavonoids in fruit might be the result of complex changes in the expression of structural/biosynthetic and regulatory genes involved in their metabolism. Therefore, in this work, the expression of structural genes involved in flavonoid biosynthesis and regulatory genes like TFs controlling this pathway was studied in parallel with the content of the major flavonoids during fruit development and senescence. Next, the interplay between ethylene and flavonoid accumulation in *Cistus* fruit was investigated. Lastly, a *Cistus* flavonoid hydroxylase (F3′5′H) was functionally characterized.

## Materials and Methods

### Plant Material

**FIGURE 2:**
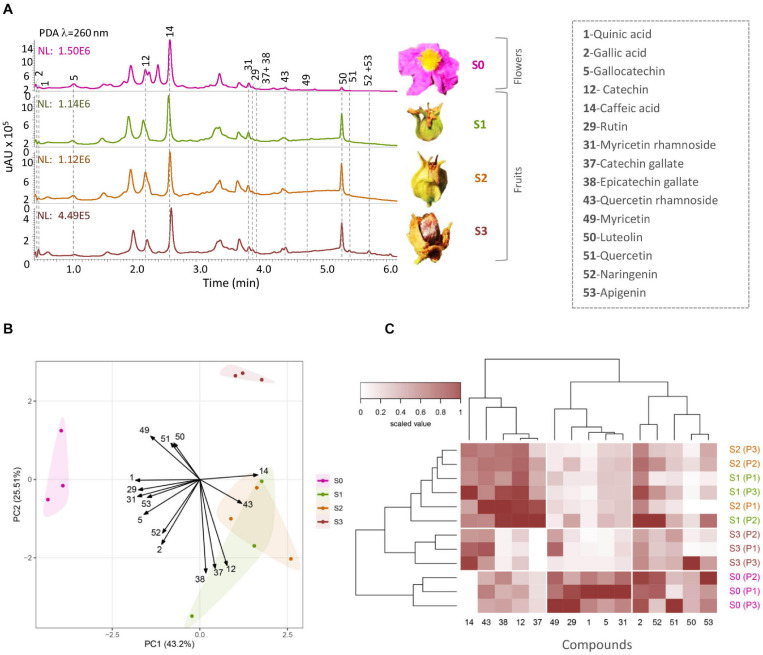
Metabolic profiling of *Cistus* flower (S0) and three fruit stages (S1–S3). **(A)** HPLC/DAD elution profiles at *λ* = 260 nm; notable peaks are denoted in the legend on the side. The *Y*-axis scales are different for each of the chromatograms. **(B)** Biplot of principal component analysis performed on zero-centered and unit-scaled compound quantity data. The samples are colored by stage (S0–S3) as shown in the legend, and these groups are further encircled by a convex hull of the same color. Variable loadings are indicated by arrows and were scaled by multiplying with five prior graph constructions so they could be seen more readily. **(C)** Heat map of zero-centered and unit-scaled compound quantity data. The samples (in rows) are arranged according to hierarchical cluster analysis (Ward’s method of cluster agglomeration) constructed using Euclidean distances (left tree), and the metabolites (in columns) are arranged according to hierarchical cluster analysis (cluster agglomeration using complete linkage) constructed using Spearman correlation distances (top tree).

*Cistus creticus* L. subsp. *creticus* plants were grown in the experimental field of the School of Agriculture, Aristotle University of Thessaloniki, located in the area of Thermi, Thessaloniki, Greece (N 40.536247° and E 22.993830°). Whole mature flowers and fruits of three different developmental stages were collected from three groups comprising five individual plants, and each group represented a biological replicate. Fruits of three developmental stages were distinguished based on the size and color of sepals and valves (Figure 2A). Small-sized half-expanded fruits with green capsules and sepals were marked as stage 1 (S1). Stage 2 (S2) was composed of fully expanded fruits with the sepals starting to change color and detached from valves, while stage 3 (S3) comprised fully colored ripe fruits. Flowers were appointed as the control organ and marked as stage 0 (S0). Upon harvest, all samples were frozen in liquid nitrogen and kept at −80°C until further use.

### Reagents and Standards

MS-grade acetonitrile and formic acid were purchased from Merck (Darmstadt, Germany). Phenolic standards were supplied by Sigma Aldrich (Steinheim, Germany) and TransMIT PlantMetaChem (Giessen, Germany). Ultrapure water was generated using TKA MicroPure water purification system, 0.055 μS/cm (Thermo Fisher, Bremen, Germany). Syringe filters (25 mm, PTFE membrane, pore size 0.45 μm) were purchased from Agilent Technologies (Econofilter, Palo Alto, CA, United States).

### UHPLC–MS Orbitrap Identification of Phenolics in *Cistus* Fruit

For chemical analyses, the frozen plant material was powdered in mortars and pestles and subsequently lyophilized. Around 100 mg of each sample was extracted with 1 ml of 99.8% methanol (AppliChem, United States) by 1 min of vortexing and subsequent sonification in ultrasonic bath (RK100, Bandelin, Berlin, Germany) for 20 min. The samples were centrifuged for 10 min at 10,000 *g*, and the supernatants were filtered. The samples were kept at 4°C until analyses. All extractions were performed in triplicate.

Chromatographic separations of compounds in methanol extracts of *Cistus* flowers (S0) and fruit (S1–S3) were performed using an Accela 600 ultrahigh-performance liquid chromatography (UHPLC) system coupled to a linear ion trap-OrbiTrap hybrid mass spectrometer (LTQ OrbiTrap MS) (Thermo Fisher Scientific, Bremen, Germany). All chromatographic and MS settings [heated electrospray ionization (HESI) and the other MS parameters] were the same as in Banjanac et al. (2017). The injection volume was 5 μl.

The mass spectrometer (MS) was operated in negative ionization mode, and MS spectra were acquired by full-range acquisition covering 100–1,500 *m/z*. The resolution was set at 30,000 for full-scan (FS) analysis, which was employed to detect the monoisotopic masses of unknown compounds. Fragmentation pathways were proposed by multistage mass spectrometry (MS^n^). The ions of interest were isolated in the ion trap with an isolation width of 5 ppm and activated with 35% collision energy (cE) levels. FS analysis was employed, while Xcalibur software (version 2.1) was used for instrument control and data analysis.

### UHPLC/DAD/(±)HESI-MS/MS Quantification of Phenolics in *Cistus* Flowers and Fruits

UHPLC/diode array detector (DAD)/(±)HESI–MS/MS method was developed for the separation, identification, and quantification of targeted phenolic compounds in samples (methanol extracts) of *Cistus* flowers and fruits. Dionex Ultimate 3000 UHPLC system (Thermo Fisher Scientific, Bremen, Germany) was equipped with a DAD and connected to a triple–quadrupole MS (TSQ Quantum Access Max, Thermo Fisher Scientific, Basel, Switzerland). Elution was performed at 40°C on Syncronis C18 column (100 mm × 2.1 mm) with 1.7-μm particle size (Thermo Fisher Scientific, Bremen, Germany). The mobile phase consisted of (A) water + 0.1% formic acid and (B) acetonitrile, which were applied in the gradient elution previously described in Mišić et al. (2015), and with a flow rate of 0.4 ml min^–1^. Acquisition of UV spectra was performed at *λ* = 260 and 320 nm, and the injection volume was set to 5 μl. The HESI source of MS was operated with a vaporizer temperature of 350°C, while the ion source settings were as follows: spray voltage, 3,510 V; sheet gas (N2) pressure, 28 AU; ion sweep gas pressure, 0.0 AU, and auxiliary gas (N2) pressure, 4 AU; capillary temperature, 270°C; and skimmer offset, 0 V. Selected reaction monitoring (SRM) experiment was conducted for the quantification of targeted phenolics, using argon as the collision gas and cE of 30 eV. The parameters of the adopted SRM UHPLC/DAD/(−)HESI–MS^2^ method are presented in Supplementary Table 1, including the major MS^2^ fragments and *λ*_max_ values of targeted compounds. Instrument control, data acquisition, and processing analysis were performed using Xcalibur software (version 2.2). The phenolics were quantified based on the calibration curves of commercial standards: gallic acid, caffeic acid, quinic acid, rutin hydrate, myricetin, luteolin, quercetin, naringenin, apigenin, catechin, epicatechin, gallocatechin, epigallocatechin, epigallocatechin gallate, and gallocatechin gallate. Myricetin 3-*O*-rhamnoside was quantified relatively using the calibration curve of myricetin. The total amount of each targeted compound is expressed as μg 100 mg^–1^ dry weight (DW).

### Ethylene Production and Respiration Rates

Air samples were taken after closing the fruit of all stages in 0.5-L glass jars for 1 h, according to Koukounaras et al. (2010). CO_2_ concentration was measured by injecting the gas samples into a stream of N_2_ carrier gas flowing through a CO_2_/O_2_ analyzer (model Combo 280, David Bishop Instruments, United Kingdom), while ethylene concentration was measured by injecting the gas sample into a Varian 3300 gas chromatographer (Varian Instruments, Walnut Creek, CA, United States) equipped with a flame ionization detector. The ethylene production and respiration rates were expressed as μl C_2_H_4_ kg^–1^ h^–1^ and mg CO_2_ kg^–1^ h^–1^, respectively.

### Selection of Candidate Genes and qPCR Analysis

Putative candidate genes involved in the flavonoid pathway were identified by a comparative analysis of *C. creticus* fruit transcriptome BLAST search against the NCBI public database. Although the BLAST search resulted in several hits for some genes involved in flavonoid and ethylene pathway, only the full-length sequences retrieved from *C. creticus* fruit transcriptome were selected for this study. Based on the detected sequences, highly specific primer pairs for qPCR were designed using NCBI Primer-BLAST^1^ (Supplementary Table 2). The sequences of characterized *CcTTG1* and *CcSPBPA/B* from *Cistus* transcriptome cDNA library (Falara et al., 2008; Ioannidi et al., 2016) have GenBank accession numbers as follows: *CcTTG1*: KT892927, *CcSPLA*: KU145276, *CcSPLB*: KU041720, and CcF3′5′H: MT707661.

For the analysis of ethylene biosynthesis, total RNA isolation was performed from *C. creticus* flowers and the three developmental stages of fruit in biological triplicates using Sigma Spectrum Kit (Sigma Aldrich, Germany), with a slight modification of the manufacturer’s instructions, namely, RNA was extracted from 50 mg of tissue; the volume of lysis buffer used was 1.5 ml per sample, whereas the volume of binding buffer was 3 ml per sample. To remove traces of genomic DNA, the RNA samples (1 μg) were treated with DNase I (Fermentas, Vilnius, Lithuania) in a final reaction volume of 10 μl. RNA quality was confirmed using NanoDrop 2000C Spectrophotometer (Thermo Scientific, United States), and their integrities were assessed by agarose gel electrophoresis.

First-strand cDNA was synthesized from 1 μg of total RNA using the SuperScript^TM^ III Reverse Transcriptase (Thermo Fisher Scientific) following the manufacturer’s instructions. The expression of genes was analyzed by real-time PCR using Light Cycler QuantStudio 3 (Thermo Fisher Scientific) and KAPA SYBR^®^ Fast qPCR Master Mix (2X) Universal (KAPA Biosystems, United States). The general thermocycler conditions were 95°C for 4 min and 40 cycles of 95°C for 10 s, 64°C for 20 s, and 72°C for 15 s. The relative expression values were normalized against elongation factor-1α (EF062868.1) as the endogenous control and calculated by the 2^–ΔΔCt^ method (Livak and Schmittgen, 2001). The data represent the means ± SD of triplicates.

### Phylogenetic Analysis

The phylogenetic analyses were performed as previously described in Aničić et al. (2020). Flavonoid-related protein sequences of MYB and nucleotide sequences of bHLH TFs were aligned using Clustal W within the software package MEGA 6.0. Gblocks v0.91b, with default parameters, was utilized to select highly conserved blocks of alignment positions. The maximum likelihood method within MEGA version 6.0 with default settings (NNI heuristic method, BioNJ initial tree, JTT model for MYB tree, and Tamura-Nei model for bHLH tree and 1,000 bootstrap replicates) was used to conduct the phylogenetic analyses of the conserved blocks.

### Functional Analysis of Putative F3′5′H by Heterologous Expression in *Saccharomyces cerevisiae*

Multiple alignment with ClustalW was used to identify the consensus of flavonoid hydroxylase genes. Known and publicly available characterized protein sequences from various plant species coding for functional F3′H and/or F3′5′H were aligned. The obtained consensus sequence served as a template in local tblastn against RNA sequencing available for *C. creticus* fruit and leaves to identify candidate genes for hydroxylases. The primers used for the PCR amplification of putative full-length genes from *Cistus* cDNA were designed in Primer3Plus online tool and were for c15585 forward: ATGGTGGAAACACTGACTCCC and reverse: TTAGGAAACATAAGCACCCGGC. Leaves of the second stage (S2) of *C. creticus* (Falara et al., 2008) were used for total RNA isolation using Spectrum Plant total RNA kit (Sigma), according to the manufacturer’s protocol. Subsequently, cDNA was synthesized with SuperScript III Reverse Transcriptase (Invitrogen) using a 1:1 mix of oligo(dT)_12__–__18_ and random primers. PCR reaction was performed in a 5-μl final volume using Q5 high-fidelity polymerase (NEB, Ipswich, MA, United States), according to the following program: initial denaturation at 98°C for 10 s, followed by 35 cycles including 98°C for 10 s, 62°C for 30 s, and 72°C for 1 min and a single cycle of a 2-min final extension at 72°C. pGEM T-Easy vector (Promega, Madison, WI, United States) was used for the cloning of the PCR-amplified product. After confirmation of selected clones by sequencing, the genes were subcloned in pYES2 yeast expression vector (Invitrogen) using the same set of primers, with the addition of *Eco*RI/*Xho*I restriction sites in their 5′ ends. The resulting plasmids were transformed into yeast strain INVSc1 (Invitrogen) using LiAc method (Gietz et al., 1992), and positive yeast colonies were verified through PCR. A 5-ml SD-URA liquid pre-culture of a single transformed yeast colony was grown in a selective SD-URA plate containing 20 g l^–1^ glucose and was incubated at 30°C and 200 rpm for 24 h. The propagated cells were collected and re-dissolved into 30-ml SD-URA medium containing 20 g l^–1^ galactose as a carbon source and for induction of protein expression. For functional characterization experiments, naringenin or dihydrokaempferol dissolved in dimethyl sulfoxide (DMSO) was separately added into the culture to a final concentration of 5 μM. The yeast culture was incubated at 30°C for 36 h. A liquid/liquid extraction of the yeast products was performed by addition of 1:1 ethyl acetate (v:v), sonication for 15 min, and centrifugation for 5 min at 11,000 × *g* for three times. The ethyl acetate fractions were collected and evaporated in an EZ-2 ENVI Genevac (GeneVac, Ipswich, United Kingdom). The crude residue was re-dissolved in 150 μl of 80% methanol (v:v), filtered through 0.22-μm polytetrafluoroethylene membrane filters into 1.5-ml glass vials, and injected directly to ultra-performance liquid chromatography (UPLC)–DAD and/or UPLC–tandem mass spectrometry (MS/MS) instruments.

### UPLC–PDA and UPLC–QTOF MS Analyses of Yeast Products

Yeast extracts were analyzed on a 1290 Infinity Binary UPLC (Agilent; Santa Clara, CA, United States) equipped with photodiode array (PDA) using a modified method described previously by Rafique et al. (2016). Separation of the compounds was achieved on a Machery and Nagel Nucleodur C18ec column (4.6 μm, 250 mm × 4 mm) set at 25°C. The gradient consisted of two solvents containing 1% phosphoric acid: solvent A was water and solvent B was acetonitrile. The analysis was performed following the chromatographic conditions, namely: 100% A to 50% A in 25 min, plateau of 3 min, up to 100% A in 7 min, and final plateau of 5 min with a flow rate of 1 ml min^–1^ and monitoring at 222 and 280 nm. In order to identify naringenin, eriodictyol, dihydrokaempferol, dihydroquercetin, and dihydromyricetin, external standards were injected in a concentration of 1 mM.

A Waters Acquity UPLC coupled *via* an electrospray ionization (ESI) interface to a Synapt HDMS QTOF MS (Waters, Manchester, United Kingdom) operating in W-mode and controlled by MassLynx 4.1 was used. Both LC and MS parameters were previously described (Shahaf et al., 2013; Arapitsas et al., 2014). Yeast extracts were chromatographically separated using an ACQUITY UPLC 1.8 μm, 2.1 mm × 150 mm HSS-T3 column (Waters, Manchester, United Kingdom), thermostated at 40°C. Mobile phase [0.1% (v/v) formic acid in water (A) and 0.1% in methanol (B)] was eluted with a flow rate of 0.28 ml min^–1^, adopting the multistep linear gradient previously described by Arapitsas et al. (2014). The injection volume was 5 μl. Mass spectrometric data were collected in negative ESI mode over a mass range of 50–2,000 amu, with a scan duration of 0.4 s in centroid mode. The source parameters, as well as transfer cE and trap cE, were set as previously reported (Arapitsas et al., 2014). Annotation of compounds in yeast extracts was performed by comparing retention times and mass spectra (mass difference less than 5 ppm, isotopic distribution, and minimum three *m/z* ions) to those of the standards and based on internal database (Shahaf et al., 2013). In cases when standards were not available (pentahydroxyflavanone), tentative identification was made by using spectral features and literature data.

### Statistical Analysis

Statistical analysis was performed using R Software (R Core Team, 2018) by applying the package stats for hierarchical clustering (HCA) and principal component analysis (PCA), gplots (Warnes et al., 2016) for heat map generation, corrplot for visualization of correlation matrices (Wei and Simko, 2017), and ggplot2 for data visualization (Wickham, 2016). HCA and PCA of metabolomics data were performed after cantering the data to 0 and scaling to unit variance. The expression data were not scaled or centered prior to performing the mentioned methods since these were already on a relative scale (log2 fold change). HCA was performed based on Euclidean distances with cluster agglomeration using Ward’s (Ward, 1963) minimum variance method. When the dissimilarity matrix was based on correlation distances (1 – cor), cluster agglomeration was performed using complete linkage. The details on dissimilarities used for HCA are provided in the legends of the respective figures. One-way analysis of variance was conducted for both metabolomics (absolute quantitates) and expression data (log2 fold change), followed by a Tukey *post hoc* test.

## Results and Discussion

### Identification of Phenolic Compounds in *Cistus* Fruit

To our knowledge, the current study is the first attempt to characterize the phytochemicals of the flowers and fruits of any *Cistus* species. Previous investigations have been primarily focused on the leaves or aerial parts (Vogt et al., 1987; Demetzos et al., 1989, 1990; Danne et al., 1993; Kreimeyer et al., 1997; Pomponio et al., 2003; Barrajón-Catalán et al., 2011; Tomás-Menor et al., 2013; Papaefthimiou et al., 2014; Maggi et al., 2016). UHPLC–MS Orbitrap analysis identified 53 compounds in the flowers and fruits of *C. creticus* subsp. *creticus*, which belong to phenolic acids, flavonoids and their derivatives, and quinic acid (Table 1). The peak numbers, compound names, molecular formulas, calculated and exact masses ([M−H]^–^, *m/z*), retention times (*R*_t_, min), mass accuracy errors, as well as major MS^2^, MS^3^, and MS^4^ fragment ions are summarized in Table 1.

**TABLE 1:** UHPLC-MS/MS Orbitrap metabolic fingerprinting of methanol extracts of *Cistus creticus* spp. *creticus* flowers (S0) and leaves of three developmental stages (S1–S3).

No	*t*_R_, min	Compound name	Molecular formula, [M–H]^–^	Calculated mass, [M–H]^–^	Exact mass, [M–H] ^–^	Δ ppm	MS^2^ fragments, (% base peak)	MS^3^ fragments, (% base peak)	MS^4^ fragments, (% base peak)	S0	S1	S2	S3
(1)	1.36	Quinic acid*^*a*^*	C_7_H_11_O_6_**^–^**	191.05611	191.05519	4.82	173(40), 173(10), 127(20), **111**(100), 93(20)	93(10), 83(10), 81(10), 67(100)	–	+	+	+	+
(2)	2.73	Gallic acid*^*a*^*	C_7_H_5_O_5_**^–^**	169.01425	169.01360	3.85	**125**(100)	107(100)	–	+	+	+	+
(3)	2.76	Gallic acid hexoside 1	C_13_H_15_O_10_**^–^**	331.06707	331.06549	4.77	294(10), **169**(100), 125(5)	**125**(100)	107(100), 81(10)	+	+	+	+
(4)	4.31	Gallic acid hexoside 2	C_13_H_15_O_10_**^–^**	331.06707	331.06600	3.23	**169**(100), 125(5)	**125**(100)	110(10), 97(30), 81(100), 53(30)	+	+	+	+
(5)	4.63	Gallocatechin*^*a*^*	C_15_H_13_O_7_**^–^**	305.06668	305.06540	4.20	261(50), 221(70), 219(70), **179**(100), 165(35)	**164**(100), 151(40), 135(30)	120(100), 108(20)	+	+	+	+
(6)	4.64	Prodelphinidin dimer B type 1	C_30_H_25_O_14_**^–^**	609.12498	609.12268	3.78	591(10), 483(10), **441**(100), 423(60), 305(30)	**423**(100)	405(20), 355(5), 297(100), 283(80), 255(20)	+	+	+	+
(7)	4.77	Dihydroxybenzoic acid hexoside 1	C_13_H_15_O_9_**^–^**	315.07216	315.07063	4.86	**153**(100), 152(50), 109(15), 108(10)	**109**(100)	123(25), 109(10), 85(10), 81(100)	+	+	+	+
(8)	5.02	Gallic acid hexoside 3	C_13_H_15_O_10_**^–^**	331.06707	331.06591	3.50	**313**(100), 211(10), 169(30), 168(80), 125(25)	193(50), **151**(100), 125(80)	123(100), 107(90), 95(65)	+	+	+	+
(9)	5.20	Prodelphinidin trimer B type	C_45_H_37_O_21_**^–^**	913.18328	913.17957	4.06	745(30), **727**(100), 609(25), 423(20), 305(10)	709(30), **559**(100), 541(30), 421(10), 305(90)	541(100), 515(10), 421(10), 391(50)	+	+	+	+
(10)	5.37	Prodelphinidin dimer A type	C_30_H_23_O_14_**^–^**	607.10933	607.10748	3.05	589(20), 579(30), **439**(100), 305(40), 301(15)	421(60), 313(70), **301**(100), 261(20), 243(30)	283(15), 273(15), 257(100), 215(20), 175(70)	+	+	+	+
(11)	5.37	Prodelphinidin dimer B type 2	C_30_H_25_O_14_**^–^**	609.12498	609.12244	4.17	591(5), 483(10), **441**(100), 423(60), 305(25)	**423**(100)	405(20), 355(5), 297(100), 283(80), 255(20)	+	+	+	+
(12)	5.45	Catechin*^*a*^*	C_15_H_13_O_6_**^–^**	289.07176	289.07065	3.84	271(5), **245**(100), 205(40), 179(15), 125(5)	227(30), **203**(100), 187(25), 175(10), 161(20)	188(70), 185(20), 175(100), 161(40), 157(10)	+	+	+	+
(13)	5.57	Vanillic acid hexoside	C_14_H_17_O_9_**^–^**	329.08781	329.08649	4.01	**167**(100)	**152**(100), 123(70), 108(20)	124(5), 108(100)	+	+	+	+
(14)	5.72	Caffeic acid*^*a*^*	C_9_H_7_O_4_**^–^**	179.03498	179.03448	2.79	**135**(100), 117(10), 91(20), 59(15)	107(100), 59(50)	–	+	+	+	+
(15)	5.80	Galloyl-HHDP-hexose 1	C_27_H_21_O_18_**^–^**	633.07334	633.07098	3.73	613(5), 481(20), 463(10), **301**(100), 275(5)	301(20), 284(30), **257**(100), 229(60), 185(40)	240(5), 229(100), 213(20), 201(10), 185(70)	+	+	+	+
(16)	5.85	Hydroxybenzoic acid hexoside	C_13_H_15_O_8_**^–^**	299.07724	299.07593	4.38	**137**(100)	93(100)	–	+	+	+	+
(17)	5.91	Digalloyl hexose	C_20_H_19_O_14_**^–^**	483.07803	483.07654	3.08	331(20), 313(20), **271**(100), 211(10), 169(10)	**211**(100), 169(15)	168(100), 124(25)	+	+	+	+
(18)	5.94	Gallic acid pentoside	C_12_H_13_O_9_**^–^**	301.05651	301.05515	4.52	283(50), 169(40), **168**(100), 150(10), 125(20)	**150**(100), 124(15)	122(100), 106(5), 94(15), 82(10)	+	+	+	+
(19)	5.98	Galloyl-HHDP-hexose 2	C_27_H_21_O_18_**^–^**	633.07334	633.07019	4.98	611(15), 602(10), 541(5), 463(5), **301**(100)	301(25), 284(30), **257**(100), 229(70), 185(40)	240(5), 229(100), 213(20), 201(10), 185(70)	+	+	+	+
(20)	5.99	Dihydroxybenzoic acid hexoside 2	C_13_H_15_O_9_**^–^**	315.07216	315.07059	4.98	**153**(100), 135(10), 109(10)	**135**(100), 109(50)	91(100)	+	+	+	+
(21)	6.11	Prodelphinidin dimer B type 3	C_30_H_25_O_13_**^–^**	593.13006	593.12775	3.89	467(15), **425**(100), 407(30), 289(20), 285(20)	**407**(100), 281(5), 273(10)	389(30), 297(30), 285(100), 243(70)	−	+	+	+
(22)	6.15	Trigalloyl hexose	C_37_H_23_O_18_**^–^**	635.08899	635.08698	3.16	541(5), 483(5), **465**(100)	447(5), **313**(100), 295(10), 235(10), 169(30)	295(20), 253(25), 241(30), 169(100), 125(15)	+	+	+	+
(23)	6.23	Procyanidin dimer B type 1	C_30_H_25_O_12_**^–^**	577.13515	577.13318	3.41	451(30), **425**(100), 407(40), 289(20), 287(10)	**407**(100), 381(5), 287(5), 273(10)	389(30), 297(30), 285(100), 281(90)	−	+	+	+
(24)	6.26	Aromadendrin 7-*O*-hexoside	C_21_H_21_O_11_**^–^**	449.10894	449.10764	2.89	288(15), **287**(100), 269(40), 259(40)	**259**(100), 243(15), 201(5)	241(30), 215(100), 173(35), 125(60)	−	+	+	+
(25)	6.33	Procyanidin dimer B type 2	C_30_H_25_O_12_**^–^**	577.13515	577.13300	3.73	451(20), **425**(100), 407(35), 289(20), 287(10)	**407**(100), 381(10), 273(10)	389(40), 297(40), 285(100), 243(75)	−	+	+	+
(26)	6.36	Myricetin 3-*O*-hexoside	C_21_H_19_O_13_**^–^**	479.08311	479.08170	2.94	317(60), **316**(100)	287(30), **271**(100), 179(40)	271(15), 243(100), 227(30)	+	+	+	+
(27)	6.40	Ellagic acid pentoside	C_19_H_13_O_12_**^–^**	433.04125	433.04004	2.79	**301**(100), 300(80)	301(95), 284(25), **257**(100), 229(70), 185(40)	229(70), 213(30), 201(15), 185(100)	+	+	+	+
(28)	6.43	Tetragalloyl hexose	C_34_H_27_O_22_**^–^**	787.09995	787.09784	2.68	635(20), **617**(100), 573(5), 465(10), 447(5)	573(80), **465**(100), 447(60), 421(15), 403(60)	447(20), 313(100), 295(15), 235(10), 169(20)	+	+	+	+
(29)	6.47	Quercetin 3-*O*-(6″-rhamnosyl)glucoside (Rutin)*^*a*^*	C_27_H_29_O_16_**^–^**	609.14611	609.14337	4.50	343(5), **301**(100), 300(30), 271(10), 255(5)	273(25), 257(20), **179**(100), 151(75)	151(100)	+	+	+	+
(30)	6.51	Myricetin 3-*O*-pentoside	C_20_H_17_O_12_**^–^**	449.07255	449.07117	3.07	317(20), **316**(100)	287(30), **271**(100), 179(30), 151(10)	271(10), 243(100), 227(40), 215(15)	+	+	+	+
(31)	6.56	Myricetin 3-*O*-rhamnoside	C_21_H_19_O_12_**^–^**	463.08820	463.08649	3.69	317(50), **316**(100)	287(30), **271**(100), 179(40)	271(15), 243(100), 227(30)	+	+	+	+
(32)	6.59	Procyanidin dimer B type 3	C_30_H_25_O_12_**^–^**	577.13515	577.13293	3.85	451(20), **425**(100), 407(40), 289(20), 287(10)	**407**(100), 381(5), 273(10)	389(30), 297(30), 285(100), 243(75)	−	+	+	+
(33)	6.61	Kaempferol 7-*O*-(6″-rhamnosyl)hexoside	C_27_H_29_O_15_**^–^**	593.15119	593.14972	2.48	327(5), 286(10), **285**(100), 257(5)	267(40), **257**(100), 241(30), 229(50), 163(20)	255(10), 239(30), 229(100), 213(20), 163(60)	+	+	+	+
(34)	6.68	*p*-Coumaric acid*^*a*^*	C_9_H_7_O_3_**^–^**	163.04007	163.03951	3.43	**119**(100)	119(60), 101(20), 93(25), 91(100), 72(10)	–	+	+	+	+
(35)	6.74	Quercetin 3-*O*-pentoside	C_20_H_17_O_11_**^–^**	433.07763	433.07651	2.59	343(5), 301(80), **300**(1000)	**271**(100), 255(60), 179(10), 151(10)	243(100), 227(80), 215(20), 199(20)	+	+	+	+
(36)	6.75	Ellagic acid*^*a*^*	C_14_H_5_O_8_**^–^**	300.99899	300.99765	4.45	284(40), 271(60), **257**(100), 229(85), 185(40)	**229**(100), 213(20), 185(85)	201(100), 185(95), 157(30), 145(20), 129(10)	+	+	+	+
(37)	6.78	Catechin gallate*^*a*^*	C_22_H_17_O_10_**^–^**	441.08272	441.08200	1.63	331(10), **289**(100), 271(10), 169(25)	271(5), **245**(100), 205(40), 179(20)	227(20), 203(100), 187(20), 175(10), 161(20)	+	+	+	+
(38)	6.84	Epicatechin gallate*^*a*^*	C_22_H_17_O_10_**^–^**	441.08272	441.08121	3.42	331(5), **289**(100), 271(15), 169(20)	271(10), **245**(100), 205(40), 179(15)	227(15), 203(100), 187(25), 175(10), 161(25)	+	+	+	+
(39)	6.95	Quercetin 3-*O*-(6″-malonyl)hexoside	C_24_H_21_O_15_**^–^**	549.08859	549.08636	4.06	**505**(100)	463(30), 445(5), **301**(100), 300(50)	273(15), 257(15), 179(100), 151(85)	+	+	+	+
(40)	7.04	Kaempferol 3-*O*-glucoside (Astragalin)*^*a*^*	C_21_H_19_O_11_**^–^**	447.09329	447.09186	3.20	327(20), 285(80), **284**(100), 255(10)	**255**(100), 227(10)	227(100), 211(60)	−	+	+	+
(41)	7.07	Dihydromyricetin	C_15_H_11_O_8_**^–^**	319.04594	319.04428	4.04	301(80), **275**(100), 233(30), 217(35), 193(45)	**257**(100), 247(40), 233(70), 217(50)	239(100), 229(20), 215(30)	+	+	+	+
(42)	7.09	Dihydroquercetin	C_15_H_11_O_7_**^–^**	303.05103	303.04974	4.26	**285**(100), 275(40), 259(70), 221(90), 179(50)	267(25), 257(65), **241**(100)	223(100), 213(75), 197(30), 185(25)	+	+	+	+
(43)	7.17	Quercetin 3-*O*-rhamnoside (Quercitrin)*^*a*^*	C_21_H_19_O_11_**^–^**	447.09329	447.09180	3.33	**301**(100), 300(35), 284(20)	273(25), 257(20), **179**(100), 151(75)	151(100)	+	+	+	+
(44)	7.25	Myricetin 3-*O*-(6″-malonyl)hexoside	C_24_H_21_O_15_**^–^**	563.10424	563.10229	3.46	**521**(100)	479(20), 317(30), **316**(100), 179(10)	287(30), 271(100), 179(40)	+	+	+	+
(45)	7.44	Cinnamoyl-digalloyl-hexose	C_29_H_25_O_15_**^–^**	613.11989	613.11707	4.60	466(10), **465**(100)	447(5), **313**(100), 295(10), 169(10)	295(10), 169(100), 151(5), 125(10)	+	+	+	+
(46)	7.45	Dihydrokaempferol (Aromodedrin)	C_15_O_11_O_6_**^–^**	287.05611	287.05484	4.42	269(10), **259**(100), 243(15), 201(10)	241(25), **215**(100), 173(30), 151(20), 125(65)	200(25), 187(10), 173(100), 158(15)	+	+	+	+
(47)	7.47	Kaempferol 7-*O*-(6″-*p*-coumaroyl)hexoside	C_30_H_25_O_13_**^–^**	593.13006	593.12799	3.49	447(10), 307(5), 286(15), **285**(100), 227(5)	**257**(100), 241(50), 229(40), 213(30), 151(70)	255(10), 239(30), 229(100), 163(40)	+	+	+	+
(48)	7.51	Pentahydroxyflavanone	C_15_H_11_O_7_**^–^**	303.05103	303.04919	4.95	**285**(100), 259(30), 217(35), 168(65), 141(40)	267(20), **257**(100), 241(80)	239(25), 229(100), 213(90), 189(40)	+	+	+	+
(49)	7.64	Myricetin*^*a*^*	C_15_H_9_O_8_**^–^**	317.03029	317.02890	4.38	299(10), 273(35), **207**(100), 163(95)	**179**(100), 151(15)	151(100)	+	+	+	+
(50)	8.52	Luteolin*^*a*^*	C_15_H_9_O_6_**^–^**	285.04046	285.03925	4.25	257(40), **241**(100), 217(50), 199(70), 175(70)	255(50), 227(100), 211(75), 197(35), 183(85)	–	+	+	+	+
(51)	8.57	Quercetin*^*a*^*	C_15_H_9_O_7_**^–^**	301.03537	301.03387	4.98	283(15), 271(60), 257(25), **179**(100), 151(80)	**151**(100)	107(100), 83(10)	+	+	+	+
(52)	9.31	Naringenin*^*a*^*	C_15_H_11_O_5_**^–^**	271.06120	271.05991	4.76	177(10), **151**(100)	**107**(100)	65(100)	+	+	+	+
(53)	9.35	Apigenin*^*a*^*	C_15_H_9_O_5_**^–^**	269.04554	269.04477	2.86	225(5), 177(15), **151**(100)	65(100)	–	+	+	+	+

**^*a*^Confirmed using available standards; The other compounds were identified using HRMS and MS^*n*^ data available in literature.(Dominant ions that are further fragmented (MS3 and MS4 experiments) are presented in bold). The presence or absence of compounds in samples is indicated with “+” or “−,” respectively*.*

Among phenolic acids, caffeic, 4-coumaric, gallic, ellagic acids, and their derivatives were found in the investigated samples (Table 1). Derivatives of gallic acid were especially abundant, including gallic acid glycosides (compounds 3, 4, 8, 15, 17, 18, 19, 22, 27, 28, and 43) and galloylated F3Os (5, 37, and 38). The results revealed the presence of six flavonoid subclasses in *Cistus* samples: flavanones, dihydroflavonols, flavones, flavonols, F3Os, and PAs. Flavones are represented by luteolin (50) and apigenin (53), while flavonols are represented by kaempferol derivatives (33, 40, and 47); quercetin (51) and its derivatives (29, 35, 39, and 43) and myricetin (49) and its derivatives (26, 30, 31, and 44), were abundant in the samples. Gallic acid glycosides and flavonol glycosides are present in *Cistus* species, and their fragmentations are very well described in the literature (Barros et al., 2013). As for dihydroflavonol derivatives, four compounds were identified, dihydrokaempferol (syn. aromadendrin) (46) and its 7-*O*-hexoside (24) as well as dihydromyricetin (41) and dihydroquercetin (42). Barrajón-Catalán et al. (2011) reported the existence of gallic acid, rutin [quercetin 3-O-(6′′-rhamnosyl)glucoside], and other glycosides of kaempferol, quercetin, and myricetin in *C. incanus* (syn. *C. creticus* subsp. *creticus*), which is in accordance with the present study. Previous chemical investigation of the air-dried aerial parts of *Cistus incanus* L. subsp. *tauricus* has also led to the isolation of protocatechuic and (−)-shikimic acid (Danne et al., 1993). Another phytochemical study on *Cistus* leaves revealed the presence of different flavonoid aglycones and glycosides belonging to the flavonol family (Hickl et al., 2018). Kaempferol, quercetin, and myricetin glycosides were also abundant in the aerial parts of *C. creticus* subsp. *eriocephalus* (Maggi et al., 2016).

Catechin (C, 12), gallocatechin (GC, 5), catechin 3-*O*-gallate (C3-*O*-gallate, 37), and epicatechin 3-*O*-gallate (EC3-*O*-gallate, 38) were F3Os identified in *C. creticus* subsp. *creticus* samples analyzed within the present study. Interestingly, epicatechin (EC) was not detectable, indicating that it is efficiently metabolized to respective derivatives. Previous phytochemical characterization of this species disclosed the presence of C, GC, GC-3-*O*-gallate, and the rarely occurring C-3-*O*-α-β-rhamnoside (Petereit et al., 1991). Among the F3Os, C, GC, EC, epigallocatechin (EGC), EC-3-*O*-gallate, EGC-3-*O*-gallate, and EGC-3-*O*-(4-hydroxybenzoate) were isolated from the leaves of *Cistus salvifolius* (Danne et al., 1994). *C. incanus* subsp. *incanus* and *C. monspeliensis* are also a rich source of GC and C (Pomponio et al., 2003), and C and EGC are abundant in *C. creticus* subsp. *eriocephalus* (Maggi et al., 2016).

The Orbitrap-MS^n^ analysis of *Cistus* flowers and fruits allowed the tentative identification of PAs based on their exact molecular masses in the negative ionization mode, the degree of hydroxylation in the B-ring of the F3Os, the presence of galloylation, the nature of the interflavan linkage, and the degree of polymerization. However, we were not able to distinguish between different stereoisomers. Here we follow the most often used classification of PAs and consider that C and EC are the subunits of procyanidins (PCs) and GC and EGC of prodelphinidins (PDs), respectively (Lin et al., 2014; Pešić et al., 2019), although some other classifications of PAs have also been proposed (Teixeira et al., 2016). The majority of identified PAs belong to the B-type, characterized by a single interflavan bond linkage between the monomers, usually C4 → C8 or C4 → C6. Only one PA identified in *C. creticus* samples (compound 10), showing [M−H]^–^ at *m/z* 607, belongs to the group of A-type PAs usually characterized by C2 → O7 linkages. This compound eluting at *R*_t_ = 5.37 min showed MS^2^ base peak at *m/z* 439 (Supplementary Figure 2), resulting from a specific Retro Diels–Alder (RDA) fragmentation (Yuzuak et al., 2018). Furthermore, MS^3^ base peak at *m/z* 301 was generated by the RDA fragmentation of the remainder part of the molecule with a pyrogallol functional group, while MS^4^ base peak at *m/z* 257 occurs by loss of the C_2_H_4_O group. Compounds 6 and 11 displayed pseudo-molecular ions [M−H] ^–^ with *m/z* at 609 (Supplementary Figure 3) and MS^2^ spectra with product ions *m/z* 305 indicating C and D rings quinone methide fission (QMCD), *m/z* 441 indicating a retro Diels–Alder fission in the C ring (RDAC), *m/z* 483 indicating a heterocyclic ring fission in ring C (HRFC), and *m/z* 591, which corresponds to the loss of a water molecule. MS^3^ ion *m/z* 423 is a result of a consequent loss of a water molecule from MS^2^ ion with *m/z* 441. These mass spectral data indicate that 6 and 11 are B-type PDs containing two (E)GC units: (E)GC-(4b → 8)-(E)GC.

Compound 21 showed pseudo-molecular ion [M−H] ^–^ at *m/z* 593, which indicated that it is a B-type PA formed from one (E)C unit and one (E)GC unit: (E)GC-(4b → 8)-(E)C or (E)C-(4b → 8)-(E)GC. The MS^2^ spectrum shows product ions *m/z* 289 (C or EC) indicating QMCD fission or a second fission after HRFC, *m/z* 425 indicating RDAC, and *m/z* 407 for a consequent loss of a water molecule, while *m/z* 245 corresponds to the (E)C loss of 44 molecular units (m.u.) (Table 1). Product ion with *m/z* 575 corresponded to the loss of a water molecule and *m/z* 467 to a HRFC. According to some studies (de Pascual-Teresa et al., 2000), the RDA fission occurs fundamentally in the upper subunit of the PA dimers, which indicates that compound 21 is a B-type PD formed with (E)GC in the upper subunit and (E)C in the lower subunit (Supplementary Figure 3). Dimeric PDs GC-(4α →)-GC, GC-(4α → 8)-C, and C-(4α → 8)-GC, EGC-3-*O*-gallate-(4β → 8)-GC, and EGC-3-*O*-gallate-(4β → 6)-GC have previously been recorded in rock rose (Petereit et al., 1991; Danne et al., 1993). A range of PD dimers have been isolated from *Cistus albidus* leaves, including EGC-(4b → 8)-C, GC-(4a → 8)-C, EGC-(4b → 6)-C, GC-(4a → 8)-GC, GC-(4a → 6)-C, EGC-(4b → 6)-GC, and GC-(4a → 6)-GC (Qa’Dan et al., 2003). Dimeric PDs such as EGC-(4 → 8)-EGC, EGC-3-*O*-gallate-(4 → 8)-EGC, EGC-(4 → 6)-EGC-3-*O*-gallate, EGC-3-*O*-4-hydroxybenzoate-(4 → 8)-EGC, and EGC-3-*O*-4-hydroxybenzoate-(4 → 8)-EGC-3-*O*-gallate were present in *C. salvifolius* leaves (Danne et al., 1994; Qa’Dan et al., 2011).

Compounds 23, 25, and 32 showed pseudo-molecular ions [M−H]^–^ with *m/z* 577, indicating that they are B-type PCs containing two (E)C units: (E)C-(4b → 8)-(E)C (Table 1 and Supplementary Figure 3). The MS^2^ spectra of these compounds showed fragment ions at *m/z* 289 and *m/z* 287, which indicated QMCD fission. MS^2^ fragment *m/z* 425, resulting from RDAC, went through a consequent loss of a water molecule, yielding the pseudo-molecular ion *m/z* 407. The presence of MS^2^ fragment at *m/z* 451 indicated a HRFC. PC EC-(4b → 8)-EC and C-(4b → 8)-C have previously been recorded in *C. incanus* (syn. *C. creticus* subsp. *creticus*) (Petereit et al., 1991) and *C. albidus* leaves (Qa’Dan et al., 2003).

Finally, compound 9 presented pseudo-molecular ion [M−H]^–^ with *m/z* 913, indicating that it is a B-type PD trimer made of three (E)GC subunits. The MS^2^ spectrum shows a fragmentation pattern with product ions *m/z* 609, a dimeric PD, indicating QMCD fission, *m/z* 745, corresponding to a RDAC, and *m/z* 305, corresponding to QMFG fission. Thus, we were able to conclude that this compound is of (E)GC-(4b → 8)-(E)GC-(4b → 8)-(E)GC type. In *C. incanus*, PD trimers GC-(4α → 8)-GC-(4α → 8)-C (Petereit et al., 1991) and GC-(4α → 8)-GC-(4α → 8)-GC (Danne et al., 1993) were recorded as well as GC-(4α → 6)-GC-(4α → 8)-GC and EGC-3-*O*-gallate-(4β → 8)-EGC-3-*O*-gallate-(4β → 8)-GC (Mansoor et al., 2016). PD trimers EGC-(4b → 8)-GC-(4a → 8)-C and EGC-(4b → 8)-GC-(4a → 8)-GC have previously been isolated from *C. albidus* leaves (Qa’Dan et al., 2003).

Qualitative metabolite analysis revealed that all 53 compounds were present in fruit stages S1–S3, while in *Cistus* flowers (S0) the following six compounds were not recorded: B-type PD dimer-isoform 3 (21), all three isoforms of B-type PC dimers (23, 25, and 32), aromadendrin 7-*O*-hexoside (24), and astragalin (40). Based on the obtained metabolite data, a flavonoid biosynthetic pathway in *Cistus* fruit has been proposed (Figure 1). The major flavonoid groups in *Cistus* fruit are flavones, flavonols, F3Os, and PAs. In *Cistus* fruit, at least three flavonoid biosynthetic branches starting from the flavanone intermediate naringenin (52) are present. One pathway results in the production of flavones apigenin (53) and luteolin (50), and the other proceeds *via* commonly occurring dihydroflavonols (dihydrokaempferol—46, dihydromyricetin—41, dihydroquercetin—42), leucoanthocyanidins (leucocyanidin and leucodelphinidin) to the anthocyanidins (cyanidin and delphinidin), which are further converted into F3Os and PAs, but also to some extent to anthocyanins as can be expected by the obvious pigmentation. Dihydroflavonols also give rise to flavonols kaemferol (40), quercetin (51), myricetin (49), and their glycosides in a side branch. It could be presumed that DFR shows distinct substrate specificity toward dihydroflavonols of the cyanidin and delphinidin branches while not converting dihydrokaempferol (46) as found in several plant species (e.g., Petunia), that LAR pathway overrides the ANR pathway in the biosynthesis of F3Os, and that catechin (12) is the predominant extension unit in oligomeric PAs in *Cistus* flowers and fruits. However, the dominant group of PAs in the analyzed fruit samples are PDs, indicating a tissue-specific expression of the flavonoid 3′,5′-hydroxylase (F3′5′H). The PA amounts were generally decreasing during *Cistus* fruit development, as given in the heat map in Figure 1. The amounts of some F3Os (EC, C) and PAs have been previously found to decrease in the progression of fruit ripening in grape (Boss et al., 1996), bilberry (Jaakola et al., 2002), and strawberry (Schaart et al., 2013). Astringent persimmon fruits are rich in PAs even at maturity, while in non-astringent types the content decreases during development (Akagi et al., 2009a).

### Patterns of Changes in Flavonoids Content During *Cistus* Fruit Development

Following Orbitrap-MS^n^ phytochemical characterization of *Cistus* flowers and fruits, a targeted metabolic approach was adopted to quantify the major polyphenolics in samples. The use of a highly sensitive and selective analytical technique, such as UHPLC/DAD/(-)HESI–MS^2^, was chosen to quantify and identify phenolic compounds in samples accurately. Metabolic profiling was targeted toward quinic acid (1), two phenolic acids (caffeic acid—14 and gallic acid—2), and 12 flavonoids, belonging to the group of flavanones (naringenin—52), flavones (luteolin—50 and apigenin—53), flavonols (quercetin—51, quercetin 3-*O*-rhamnoside—43, rutin—29, myricetin—49, myricetin 3-*O*-rhamnoside—31), and F3Os (catechin—12, gallocatechin—5, catechin 3-*O*-gallate—37, and epicatechin 3-*O*-gallate—38). PAs were not quantified due to the lack of available standards. The UHPLC/DAD chromatograms of *Cistus* flowers (S0) and of three developmental fruit stages (S1–S3) are presented in Figure 2A.

The peak eluting at *R*_t_ = 0.46 min and displaying the deprotonated molecule [M−H]^–^ at *m/z* 191 was identified as quinic acid (1) (Figure 2A and Supplementary Table 1). Although 1 is not a phenolic compound, it was interesting to trace its amount in *Cistus* fruits because it is involved in the regulation of the biosynthesis of aromatic compounds (Ghosh et al., 2012). This free acid is synthesized *via* the shikimate pathway and is abundant in a variety of fruits, such as papaya, pineapple, lemon, kiwi, cranberry, lingonberry, blueberry, apple, and orange (Jensen et al., 2002; Chinnici et al., 2005; Albertini et al., 2006; Erk et al., 2009; Hernández et al., 2009; Barboni et al., 2010; Ye et al., 2014). Significant amounts of 1 were found in S0 stage (flower) (∼12 μg 100 mg^–1^ DW), while it was significantly lower in fruit (stages S1–S3) (Supplementary Figure 4).

The peak eluting at *R*_t_ = 0.66 min and displaying deprotonated molecule [M–H]^–^ at *m/z* 169 was identified as gallic acid (2) (Figure 2A and Supplementary Table 1). The amount of 2 slightly decreased during *Cistus* fruit development, and in S3 stage it reached ∼4 μg 100 mg^–1^ DW (Supplementary Figure 4). Gallic acid (2) and galloylated F3Os were identified in *C. albidus*, *Cistus clusii*, *Cistus crispus*, *C. creticus*, *Cistus ladanifer*, *Cistus laurifolius*, *C. monspeliensis*, *Cistus populifolius*, and *Cistus salviifolius* leaf samples collected in Spain (Santagati et al., 2008; Barrajón-Catalán et al., 2011). In PA-rich berries, such as grapes, 2 was mainly accumulated as galloylated F3Os (Bontpart et al., 2016). The glucose ester of 2, β-glucogallin (β-G), is not only involved in the biosynthesis of hydrolysable tannins (Haslam and Cai, 1994; Niemetz and Gross, 2005) but also the donor of 2 for galloylated PAs (Liu et al., 2012). In *Cistus* fruit, 2 was present both as free and in the form of galloylated F3Os. Caffeic acid (14), with pseudomolecular ion [M–H]^–^ at *m/z* 179, eluted at *R*_t_ = 3.54 min. Trace amounts of 14 were recorded in flowers (S0 stage), while it was relatively constant in fruit stages S1 to S3, with concentrations ranging from 0.1 to 0.15 μg 100 mg^–1^ DW (Supplementary Figure 4).

The peak visible at *R*_t_ = 5.87 min, showing [M–H] ^–^ at *m/z* 301, was assigned as flavonol quercetin (51). Its amount was relatively stable in flowers and fruits of all stages, although it is usually further metabolized to different glycosides. The peak eluting at *R*_t_ = 3.95 min, with pseudo-molecular ion [M−H] ^–^ at *m/z* 609, displayed MS2 fragmentation pattern (Supplementary Table 1) characteristic for the flavonol glycoside rutin (29). The content of 29 was the highest in flowers (S0 stage) but significantly reduced in all stages of fruit (S1–S3) (Supplementary Figure 4). The amount of quercetin 3-*O*-rhamnoside (43), showing [M–H]^–^ at *m/z* 447 and eluting at *R*_t_ = 4.44 min, was not significantly changed within the analyzed *Cistus* flowers and fruits. Myricetin (49), which eluted at *R*_t_ = 4.76 min, was identified as deprotonated molecular ion [M–H]^–^ at *m/z* 317. The amount of 49 was the highest in *Cistus* flowers (S0 stage) and S3 fruit but significantly decreased in S1–S2 fruit (Supplementary Figure 4). The major flavonol in *Cistus* fruit was myricetin 3-*O*-rhamnoside (31), reaching around 23 μg 100 mg^–1^ DW in flowers (S0). As in the case of 49, its amount was severely reduced in fruit (stages S1–S3) (Supplementary Figure 4). Therefore, the tissue- and stage-specific expression of the F3′5′H to yield the precursor dihydromyricetin for flavonol formation and a rhamnosyltransferase involved in the biosynthesis of 31 can be predicted. The peak corresponding to 31 and showing [M–H]^–^ at *m/z* 463 eluted at *R*_t_ = 4.01 min.

Apigenin (53), with molecular ion [M−H] ^–^ of *m/z* 269, eluted at *R*_t_ = 5.83 min, while luteolin (50), showing [M−H] ^–^ at *m/z* of 285, eluted at *R*_t_ = 5.37 min (Figure 2A and Supplementary Table 1). The glycosides of the two flavones were not detectable in this study. Flavanone naringenin (52) (*R*_t_ = 5.71 min), showing pseudo-molecular ion [M−H] ^–^ at *m/z* of 271, was identified by the characteristic fragmentation patterns (Supplementary Table 1). The content of 50, 52, and 53 was not significantly different between *Cistus* flowers (S0) and fruits (Supplementary Figure 4). Some flavonoids, including 49, 51, 53, and kaempferol and their derivatives, are abundant in the leaves and resin of Cretan *C. creticus* subsp. *creticus* (Demetzos et al., 1989, 1990). In the present study, kaempferol was not detected in rock-rose flowers and fruits, indicating its efficient and complete conversion by glycosyltransferases. Similarly, kaempferol was not recorded in the methanol extracts of *C. creticus* and *C. monspeliensis* leaves (Hickl et al., 2018). Although a few kaempferol glycosides are identified in *Cistus* flowers and fruits (Table 1), they were present in amounts which were below the limits of quantification of the analytical procedure and were thus not quantified.

The major F3Os in *Cistus* fruits are GC (5) and C (12), while C-3-*O*-gallate (37) and EC-3-*O*-gallate (38) were less abundant. C (12) was visible in the negative ionization mode as adduct with formic acid, which was used as the mobile phase. It showed pseudo-molecular ion [M−H^+^formic acid]^–^ at *m/z* of 334 and was eluted at *R*_t_ = 2.29 min (Supplementary Table 1), and its MS^2^ fragmentation pattern was in accordance with some previous studies (Del Rio et al., 2004; Stöggl et al., 2004). Galloylated F3Os 37 and 38, visible as pseudo-molecular ions [M−H]^–^ at *m/z* of 441, were eluted at *R*_t_ = 4.03 min and *R*_t_ = 4.08 min, respectively. Their MS^2^ fragmentation profiles were the same and were characterized by the predominance of fragments [M−H-galloyl group]^–^ at *m/z* of 289 (−152 Da), matching the deprotonated (E)C and [M−H-catechin]^–^ *m/z* of 169, corresponding to deprotonated 2. Gallocatechin (5), showing [M−H]^–^ at *m/z* 305, eluted at *R*_t_ = 1.12 min and displayed MS^2^ fragmentation pattern (Supplementary Table 1). The contents of targeted F3Os varied during *Cistus* fruit development. The content of 12, 37, and 38 was the highest in fruits of S1 and S2 stages (Supplementary Figure 4). The content of 5 was the highest in flowers (S0), with a concentration of ∼40 μg/100 mg^–1^ DW, and it decreased, during fruit development, down to ∼1.9 μg/100 mg^–1^ DW in S3 phase (Supplementary Figure 4). F3Os EC, C, EGC, and GC have been previously reported for *C. incanus* (Riehle et al., 2013).

Among the analyzed phenolics, the major compounds in flowers were GC (5), myricetin 3-*O*-rhamnoside (31), C (12), gallic acid (2), rutin (29), apigenin (53), and naringenin (52). Quinic acid (1) was very abundant in flowers (Supplementary Figure 4). In fruits, C (12), GC (5), myricetin 3-*O*-rhamnoside (31), quercetin 3-*O*-rhamnoside (43), and gallic acid (2) were the most abundant compounds. The abundance of compounds 5, 31, and 29 indicates differential hydroxylase and rhamnosyltransferase activity in *Cistus* flowers and fruits.

The quantitative content of targeted metabolites obviously changed during *Cistus* fruit development, as supported by PCA analysis (Figure 2B). Not surprisingly, the chemical profile of *Cistus* flowers (stage S0) was distinctively different from that of fruit of all developmental stages (S1–S3), with PC1 accounting for 43.2% of the total variance. The main contributors to PC1 are 31, 1, 29, and 14. On the other hand, fruit stage S3 segregates from S1 and S2 in PC2, explaining 25.51% of the data variance. Stages S1 and S2 showed no visible separation in PC1 and PC2. The highly contributing compounds to PC2 are 38, 37, 12, 49, 51, and 50. The PCA indicates that stages S1 and S2 are phytochemically closer to each other than to stages S0 and S3.

For a better perception on the phytochemical correlation among flowers (S0) and the three developmental fruit stages (S1–S3), data per compound normalized to 0–1 range, are presented as a heat map (Figure 2C) with biological replicates (in rows) arranged according to HCA based on Euclidean distances and metabolites (in columns) organized according to HCA based on Spearman correlation distances (1 −cor_sp_, i.e., 100% positive correlation equals 0 and 100%, negative correlation equals 2). Flowers (stage S0) form a homogenous cluster, while fruits of different developmental stages are clustering together. However, samples belonging to stage S3 form a separate sub-cluster. On the other hand, HCA based on Spearman correlation distances (Figure 2C, top) provides a clear depiction of the targeted compound linkages. Two distinct clusters are visible. The first cluster contains the majority of F3Os (12, 37, and 38) and compounds 14 and 43. The second cluster is divided into two subclusters. The first sub-cluster consisted of flavonol aglycones and metabolites 1, 5, 29, 31, and 49, while all the rest of the compounds (2, 50, 51, 52, and 53) belonged to the second sub-cluster.

### Expression Patterns of Flavonoid Biosynthetic Genes and Related Transcription Factors During *Cistus* Fruit Development

The present study was conducted to comprehend how structural biosynthetic genes are regulated during *Cistus* fruit development and senescence to balance the synthesis for flavones, flavonols, F3Os, and PAs. Transcriptomes of *C. creticus* fruit were searched for homologs of known flavonoid pathway biosynthetic genes and related TFs. The expression patterns of the following genes in flowers (S0) and throughout the three fruit developmental stages (S1–S3) have been investigated in more detail: phenylalanine ammonia-lyase (*CcPAL1* and *CcPAL2*), cinnamate 4-hydroxylase (*CcC4H*), 4-coumarate:coenzyme A ligase (*Cc4CL1* and *Cc4CL2*), chalcone synthase (*CcCHS1* and *CcCHS2*), chalcone isomerase (*CcCHI*), flavanone-3β-hydroxylase (*CcF3H1* and *CcF3H2*), dihydroflavonol-4-reductase (*CcDFR1* and *CcDFR2*), flavonoid-3′,5′-hydroxylase (*CcF3′5′H*), flavonol synthase (*CcFLS*), leucoanthocyanidin reductase (*CcLAR1* and *CcLAR2*), anthocyanidin synthase (*CcANS*), and anthocyanidin reductase (*CcANR*) (Supplementary Figure 5).

The expression of *CcPAL1*, *CcPAL2*, *CcCHS1*, and *CcCHI* is down-regulated during fruit development and senescence (Supplementary Figure 5) which is in agreement with the metabolomics data, showing the highest naringenin (52) levels in flowers (S0) and S1 *Cistus* fruit and its decrease during further fruit development. Comparable data were observed during development of the apple fruit (Henry-Kirk et al., 2012). Conversely, the levels of *Cc4CL1* transcripts were relatively high in flowers and fruits of S1 and S2 developmental stages, and their expression decreased in S3 fruit (Supplementary Figure 5). For *CcC4H*, *Cc4CL2*, and *CcCHS2*, no apparent differences between samples were recorded, although the expression was slightly higher in flowers (S0) and S1 fruits. The enzyme *4CL* converts *p*-coumaric acid to *p*-coumaroyl-CoA. This enzyme is concurrently engaged in controlling the efflux of *p*-coumaroyl-CoA in divergent branches of the phenylpropanoid pathway as well as in promiscuously converting other hydroxycinnamic acids (caffeic and ferulic acid) in the lignin biosynthesis. Therefore, the slightly different correlation pattern of *4CL2* expression in comparison with the other structural genes of flavonoid biosynthesis could indicate its additional role in lignification of *Cistus* fruit. The transcript levels of *CcF3H1* and *CcF3*′5′*H* were relatively stable in flowers (S0) and in S1 and S2 fruits, and they significantly decreased in S3 fruit (Supplementary Figure 5). The expression of *CcF3H2* is the highest in flowers (S0) and is down-regulated during fruit development, with the lowest transcript levels recorded in S3 fruit. A similar trend was observed for *CcFLS* gene, which is responsible for converting dihydroflavonols into flavonols. Thus, both *CcF3H1* and *CcF3H2* are active in flowers, while in fruit of early developmental stages only *CcF3H1* is involved in the synthesis of flavonols and PAs. The expression of *CcDFR*, which reduces dihydroflavonols to leucoanthocyanidins, was detected throughout fruit development, and the transcript levels of both *CcDFR1* and *CcDFR2* reached their maximum in flowers (S0) and decreased during fruit development (Supplementary Figure 5). Two pathway branches are involved in the synthesis of F3Os, the *LAR* and *ANR* branches. In *Cistus* fruit, *CcANR* and *CcLAr1* and also the intermediate *CcANS* expressions are relatively stable in flowers and S1 and S2 fruits but are decreased in S3 fruit. The transcript levels of a second *LAR* candidate, *CcLAR2*, are highest in flowers (S0) and slightly drop during fruit development and ripening (S1 and S2), reaching significantly lower amounts in ripe S3 fruit (Supplementary Figure 5).

In view of the above-mentioned condition, it could be presumed that flavonoid metabolism is differently modulated in flowers and fruits in a way to complement their morphology, physiology, and function. Higher transcript levels of LBGs (*CcDFR1*, *CcDFR2*, *CcANR*, *CcANS*, *CcLAR1*, and *CcLAR2*) and, at the same time, generally lower F3Os content in flowers, when compared to fruits, led to the belief that the flavonoid pathway in flowers is most likely directed toward anthocyanin biosynthesis, the main pigments of pink *Cistus* flowers, and/or toward PAs, both not quantified in this tissue within the present study. In fruits (S1–S3), the transcript levels of *CcANR*, *CcANS*, *CcLAR1*, and *CcLAR2* followed the trend of F3Os content during their development and ripening. Based on metabolomic and transcriptomic data, it could be presumed that the LAR pathway is the predominant one so that the metabolic flux is directed toward the synthesis of 2R,3S-*trans*-flavan-3-ols (GC, C, and C-3-*O*-gallate). Although galloyled F3Os, such as GC, CG, and ECG, are recorded in *Cistus* fruits, the genes directly involved in their biosynthesis (*CcECGT* and *CcCGT*) have not been identified in the transcriptome of *C. creticus* fruit (stage S2) and were therefore not analyzed within the present study. During the maturation and ripening of bilberry (Zifkin et al., 2012), blackberry (Chen et al., 2012), and peach (Zhou et al., 2015), a decrease in the expression of ANR and LAR genes was observed similarly to the data obtained here. Furthermore, genes specific for the PA pathway, *LAR* and *ANR*, have been functionally characterized in a variety of fruit crops, such as grapevine (Bogs et al., 2005), persimmon (Ikegami et al., 2007; Akagi et al., 2009a, b), apple (Han et al., 2012; Henry-Kirk et al., 2012), strawberry (Schaart et al., 2013), and peach (Ravaglia et al., 2013). Besides the known function of LAR in the conversion of, e.g., leucocyanidin to (+)-catechin (Tanner et al., 2003), a new role in regulating the oligomerization and extension of PAs in *Medicago truncatula* has been proposed (Liu C. et al., 2016).

**FIGURE 3:**
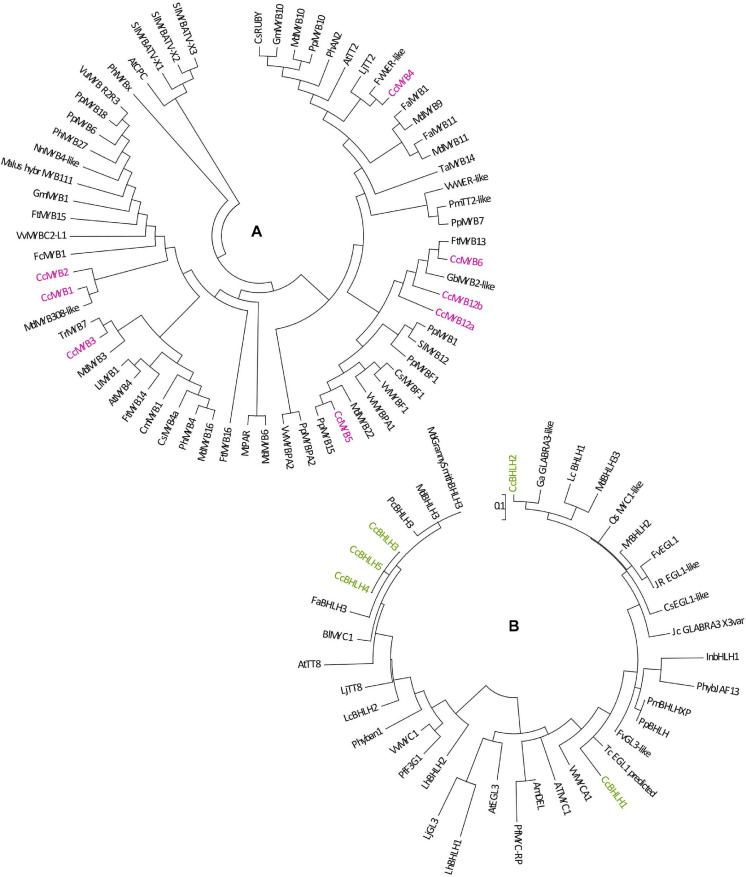
Phylogenetic trees derived from MYB and BHLH transcription factors involved in the regulation of flavonoid pathway. Phylogenetic analysis of putative MYB amino acid **(A)** and BHLH nucleotide sequences **(B)** from *Cistus* and a range of other species. Sequences were aligned using Clustal W, and a maximum likelihood tree was formed from the alignment. The scale bars represent 0.2 **(A)** and 0.1 **(B)** substitutions per site. The GenBank accession numbers are as follows: AtCPC (AAN78321.1), AtMYB4 (AT4G38620), AtTT2 (AJ299452), Ccl MYB41like (XP_024033719.1), CmMYB1 AEO27497.1, CpMYB41-like (XP_021904296.1), CsMYB20 (XP_006482113.1), CsMYB4a (ARB51599.1), CsMYB34 (XP_006488922.1), CsMYBF1 (AMH40451.1), CsRUBY (NP_001275818.1), DkMYB4 (AB503701), FaMYB1 (AF401220), FaMYB5 (AZI95727.1), FaMYB11 (JQ989282), FcMYB1 (ADK56163.1), FtMYB13 (APZ74338.1), FtMYB14 (APZ74339.1), FtMYB15 (ASK85760.1), FtMYB16 (APZ74341.1), FvWER-like (XP_011468576.1), GbMYB2-like (ACI23563.1), GmMYB1 (ACM62749.1), GmMYB10 (FJ197137), LjTT2a (AB300033), LlMYB1 (ADY38393.2), Malus_hybr MYB111 (AHG99475.1), MdMYB3 (AEX08668.1), MdMYB5-like (XP_008356551), MdMYB6 (AAZ20429.1), MdMYB9 (ABB84757.1), MdMYB10 (ACQ45201.1), MdMYB11 (AAZ20431.1), MdMYB16 (ADL36756.1), MdMYB22 (AAZ20438.1), MdMYB308-like (XP_008369485), MtPAR (HQ337434), NnMYB4-like (XP_010259378.1), PhAN2 (ABO21074), PhMYB4 (ADX33331.1), PhMYB27 (AHX24372.1), PhMYBx (AHX24371.1), PmTT2-like (XP_008238440), PpMYB6 (XP_020419855.1), PpMYB7 (ALO81018.1), PpMYB10 (ADK73605.1), PpMYB15 (ONH94094.1), PpMYB18 (ALO81021.1), PpMYBF1 (ONI28129.1), PpMYBPA2 (XM_007203070), SlMYB12 (ACB46530.1), SlMYBATV-X1 (AUG72360.1), SlMYBATV-X2 (AUG72361.1), SlMYBATV-X3 (AUG72362.1), TaMYB14 (JN049641), TaMYB5 (XP_007039783), TcMYB20 (EOY03898.1), TrMYB7 (AMB27080.1), VuMYB R2R3 (AKR80571.1), VvMYB4a (ABL61515.1), VvMYB4b (ACN94269.1), VvMYB5a (NP_001268108.1), VvMYB5b (NP_001267854.1), VvMybC2-L1 (NP_001268133.1), VvMYBF1 (ACT88298.1), VvMYBPA1 (CAJ90831.1), VvMYBPA2 (ACK56131.1), VvWER-like (XP_010646852.1). AmDEL (M84913.1), AtEGL3 (NM_001198373.2), AtMYC1 (NM_001340255.1), AtTT8 (NM_117050.3), BlMYC1 (KP245830.1), CsEGL1-like (XM_006491035.2), FaBHLH3 (JQ989284.1), FvGL3-like (XM_004298754.2), FvEGL1 (XM_004308329.2), GaGLABRA3-like (XM_017772602.1), InBHLH1 (AB232774.1), JcGLABRA3-X3var (XM_012231504.2), JrEGL1-like (XM_018952436.1), LcBHLH1 (KY302803.1), LcBHLH2 (KY302804.1), LhbHLH1 (AB222075.1), LhbHLH2 (AB222076.1), LjGL3 (AB492284), LjTT8 (AB490778.1), MdBHLH3 (HM122458.1), MdbHLH33 (DQ266451.1), Md’GrannySmith′BHLH3 (KX822759.1), MrBHLH2 (JX629462.1), PcBHLH3 (KT254006.1), PfF3G1 (AB103172.1), PfMYC-RP (AB024050.1), Phyban1 (AF260919.1), PhybJAF13 (AF020545.1), PmBHLH (XP_008238828.1), PpBHLH (XM_020564682.1), QsMYC1-like (XM_024018235.1), TcEGL1-like (XM_007040187.2), VvMYC1 (EU447172.1), and VvMYCA1 (EF193002.3).

To explain the regulatory background of *Cistus* flavonoid metabolism during fruit development, we studied the expression profile of MYB, bHLH, and WD40 TFs. MYB proteins function as direct activators of structural genes and as activators of the gene(s) encoding bHLHs (Schaart et al., 2013). Regulation of F3O and PA biosynthesis may be conditioned by the feedback interactions of MYB and bHLH components of the MBW activation complex (Chezem and Clay, 2016). In *Arabidopsis thaliana*, the MBW complex formed by MYB, bHLH, and TTG activates the genes *DFR*, *ANS*, and *ANR*, the products of which coordinate the production of PAs in the seed coat (Nesi et al., 2001; Debeaujon et al., 2003). The regulation of F3O and PA biosynthesis and accumulation has previously been studied in fruits of several species, including persimmon (Ikegami et al., 2007; Akagi et al., 2009a, b), grape (Czemmel et al., 2009; Terrier et al., 2009), and apple (Henry-Kirk et al., 2012). In grape berries development, several MYBs (*VvMYBPA1*, *VvMYBPA2*, *VvMYB5a*, and *VvMYB5b*) specifically regulate PA synthesis (Deluc et al., 2008; Bogs et al., 2007; Terrier et al., 2009). PA-specific MYB regulators (*DkMYB2* and *DkMYB4*) have also been described from persimmon fruit, which has unusually high PA levels (Akagi et al., 2009b, 2010). *DkMYB4* was found to be the specific activator of *DkANR* but not of *DkLAR* (Akagi et al., 2010). To date, PA-related MYB activators have been identified in fruits of various plant species, such as *PpMYBPA1* in nectarine (Ravaglia et al., 2013), *MdMYB9/MdMYB11* in apple (Gesell et al., 2014; An et al., 2015), *PpMYB7* in peach (Zhou et al., 2015), and *PbMYB9* in pear (Zhai et al., 2015). Transgenic tomato lines expressing *AtMYB12* TF of *Arabidopsis* under constitutive promoter exhibited an enhanced accumulation of flavonols in fruits, accompanied with the elevated expression of phenylpropanoid pathway genes involved in flavonol biosynthesis (Pandey et al., 2015). Generally, MYB factors are involved in primary and secondary metabolism; they regulate many physiological processes in plants such as cell fate and identity, development, hormone signal transduction, and response to environmental stresses (Dubos et al., 2010). To examine the patterns of PA regulation by MYBs, we traced the expression of eight MYB candidates identified in *Cistus* fruit transcriptome (*CcMYB1*, *CcMYB2*, *CcMYB3*, *CcMYB4*, *CcMYB5*, *CcMYB6*, *CcMYB12a*, and *CcMYB12b*). The expression profiles of these MYBs followed the same trend, being relatively stable in flowers (S0) and in S1 and S2 stage fruits, whereas these decreased significantly in S3 fruit (Supplementary Figure 5). To propose a putative specific function of identified *C. creticus* MYBs, a phylogenetic analysis was constructed for MYB TFs of different plants (Figure 3A). *CcMYB4* clusters closely with PA-related MYB activators, such as *AtMYB123* (*AtTT2*) in *Arabidopsis* (Nesi et al., 2001), *MdMYB9/MdMYB11* and *MdMYB6* in apple (Gao et al., 2011; Gesell et al., 2014; An et al., 2015), and others. Anthocyanin and PA-related MYB activators require specific bHLH co-activators to work, and they contain a bHLH-binding domain in the N-terminal R3-MYB repeat (Ma and Constabel, 2019). The lignin- and flavonol-activating MYBs do not have this domain. Since *Cistus CcMYB4* contains bHLH-binding domain, it could be presumed that it is most likely anthocyanin and PA biosynthesis activator. *C. creticus CcMYB5* and *CcMYB6* contain no bHLH-binding domain and are presumably involved in the activation of lignin and/or flavonol biosynthesis. They cluster close to the MYBs of the flavonol clade (Figure 3A). The same goes for *CcMYB12a* and *CcMYB12b* which, according to some previous studies on *A. thaliana* and *Solanum lycopersicum* (Mehrtens et al., 2005; Ballester et al., 2010; Pandey et al., 2015), might play an important role in regulating the flavonol pathway. *Cistus CcMYB1* and *CcMYB2* are clustered closely to grapevine *VvMYBC2L-1* (Huang et al., 2014; Cavallini et al., 2015), which falls into the group of MYB repressors. However, based on the present study, *CcMYB1* and *CcMYB2* expression in *Cistus* flowers and fruits follows the decreasing expression patterns of biosynthetic genes during the fruit’s development. Similarly, *CcMYB3* is similar to apple *MdMYB16* (Xu et al., 2017) and other R2R3-MYB repressors involved in the regulation of general phenylpropanoid and lignin biosynthetic pathway but is down-regulated during *Cistus* fruit development. Within the MYB phylogeny, most MYB repressors belong to the subgroup of R2R3-MYBs (Ma and Constabel, 2019), which is separated into two clusters, both containing the bHLH domain: (1) a general phenylpropanoid and lignin MYBs (*CcMYB3*) and (2) flavonoid-related MYBs (*CcMYB1 and CcMYB2*). Only the anthocyanin and PA repressors interact with bHLH proteins. It has been suggested that, unlike most MYB flavonoid activators, MYB repressors seem to affect the biosynthesis of multiple flavonoids due to their bHLH-binding activity (Ma and Constabel, 2019). Activator MYBs are more specific than corresponding repressor MYBs. The mechanism of action of activator and repressor MYBs is largely determined by their competition for corresponding cofactor or DNA *cis*-element. Binding of flavonoid MYB repressors with bHLH co-activators can interfere with the MBW complex (Ma and Constabel, 2019). The relative abundance of MYB activator and repressor proteins in a given cell determines the incident of promoter activation and repression events. Functional characterization of identified *Cistus* MYBs would suggest their regulatory role within the flavonoid biosynthetic pathway in fruits.

The MBW complexes might include different classes of MYBs and bHLHs with specific functions in regulating the transcription of flavonoid biosynthetic genes. Within the present study, we also examined several *Cistus* bHLH candidates, and the majority of them (*CcbHLH3*, *CcbHLH4*, and *CcbHLH5*) showed a relatively stable expression in flowers and during the early development of fruits (S1 and S2 stages) and a decreasing trend in late fruit developmental stage (S3) (Supplementary Figure 5). The exceptions are *CcbHLH1* and *CcbHLH2* since their expression did not change during fruit development. It is not strange that some *bHLH* genes show observable different transcript behavior since they are regulating different branches of the flavonoid biosynthetic pathways and possibly other metabolic pathways. Some previous studies showed that the expression of 113 *FabHLH* genes in strawberry is dependent on the variety, fruit tissues/organs, and developmental stages (Zhao et al., 2018). By comparing the nucleotide sequences of *Cistus bHLHs* and *bHLHs* from other species, the phylogenetic tree was constructed (Figure 3B). According to Montefiori et al. (2015), the bHLH TFs have been divided into two major groups, bHLH2/AN1/TT8 and bHLH/JAF13/EGL3 clades. Many members of both clades have been shown to regulate different branches of the flavonoid pathway (Montefiori et al., 2015). *Cistus* bHLH candidates *CcbHLH1* to *CcbHLH3* belong to the bHLH/JAF13/EGL3 clade (Figure 3B), together with grapevine *VvMYC1* (Hichri et al., 2010), *Arabidopsis* bHLH proteins *AtGL3* and *AtEGL3* (Payne et al., 2000), apple *MdbHLH33* (Espley et al., 2007), lychee *LcbHLH1* (Lai et al., 2016), and petunia *PhJAF13* (Quattrocchio et al., 1998), which are involved in the production of different flavonoids. The grape bHLH TFs *VvMYC1* and *VvMYCA1* were found to induce anthocyanin and PA production through the interaction with corresponding MYBs and consequent activation of the promoters of biosynthetic genes (Hichri et al., 2010). In lychee, *LcMYB1* has been identified as the key regulator of anthocyanin biosynthesis (Lai et al., 2014). On the other hand, *CcbHLH4* belongs to the bHLH2/AN1/TT8 clade, clustering close to strawberry *FabHLH3* and putative negative regulator *FabHLH3*Δ (Schaart et al., 2013), apple *MdbHLH3* (Espley et al., 2007), *VvMYC1* (Hichri et al., 2010), *Arabidopsis AtTT8* (Nesi et al., 2000), lychee *LcbHLH2* (Lai et al., 2016), *etc*. Strawberry *FabHLH3* and FabHLH3Δ are found to be involved in PA biosynthesis through the interaction with four MYBs (Schaart et al., 2013).

Similarly as MYBs and the majority of bHLHs, *Cistus* TTG candidate (*CcTTG*) showed a decreasing trend of expression during fruit development and ripening (Supplementary Figure 5). It was previously suggested that, *in planta*, TTG1 could regulate both the specific activity (i.e., interactions with other proteins or DNA) and the quantity (e.g., stability and localization) of the MBW complexes (Xu et al., 2015) and is therefore essential for flavonoid biosynthesis. Apart from its role in flavonoid biosynthesis, it is suggested that MYB-BHLH-TTG1 complex is modified by squamosal promoter-binding protein (SPBPs) to control trichome development and patterning in *C. creticus* leaves (Ioannidi et al., 2016). Overexpression of *CcSPBPA/B Cistus* genes in *Arabidopsis* leaves affected a broad range of plant developmental processes, most probably by directly binding to TTG1 (Ioannidi et al., 2016). Moreover, previous reports have shown that the elevated levels of SPBPs negatively modify TTG1-dependent physiological processes, including anthocyanin accumulation and trichome distribution of floral stems and leaves (Gou et al., 2011; Ioannidi et al., 2016). The expression patterns of the two SPBP candidates identified, *CcSPBP1* (syn. *CcSPLA*) and *CcSPBP2* (syn. *CcSPLB*), showed opposite trends in flowers (S0) and fruits (S1–S3). The expression of *CcSPBP1* was increased in stages S1–S3 when compared to S0, while the level of *CcSPBP2* transcripts gradually decreased during *Cistus* fruit development and was lowest in S3 stage (Supplementary Figure 5). These results indicate that *CcSPBP1* is most likely an isoform involved in the regulation of flavonoid metabolism in fruit, while *CcSPBP2* predominates in flowers. Negative correlations observed between *CcTTG* and *CcSPBP1* further support the proposed role of *CcSPBP1* as the MBW antagonist influencing flavonoid biosynthesis in rockrose fruit.

### Ethylene Biosynthesis and Respiration During *Cistus* Fruit Development

Distinct hormone signaling pathways are known to be implicated in the regulation of the flavonoid pathway. In this context, the regulatory roles of abscisic acid (ABA) (Lacampagne et al., 2010; Zifkin et al., 2012), jasmonate (JA) (An et al., 2015; Delgado et al., 2018), ethylene (Min et al., 2012), brassinosteroids (Zhou et al., 2018), and other hormones have been proposed.

**FIGURE 4:**
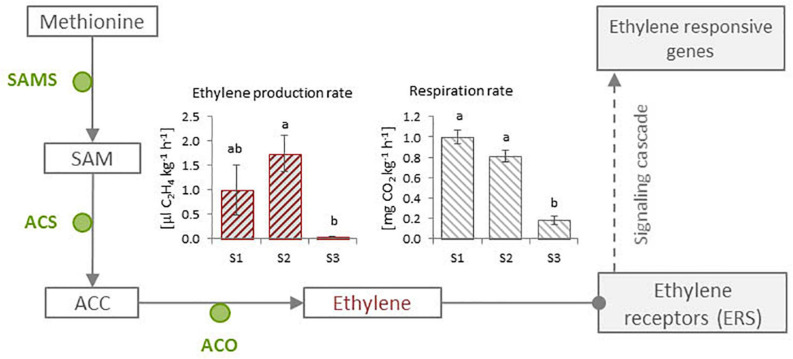
Ethylene production and respiration in *Cistus* fruits of three developmental stages (S1–S3). Bars with different letters are significantly different (*p* < 0.05) according to ANOVA *post hoc* Tukey’s test. Ethylene is synthesized from methionine (A) in a simple three-step biosynthetic pathway involving the enzymes *S*-adenosyl methionine synthetase (SAMS), 1-amino cyclopropane-1-carboxylate synthase (ACCS), and 1-amino cyclopropane-1-carboxylate oxidase (ACO). *S*-adenosyl methionine, synthesized from methionine in a reaction catalyzed by SAMS, is converted by ACCS into ACC, and then ACC is oxidized by ACO to form ethylene. Ethylene further induces a signaling cascade which activates ethylene-responsive genes.

The highest ethylene production rate was observed in *Cistus* fruits of S1 and S2 stages, which is followed by a significant reduction in S3 stage. Respiration rates gradually decreased during fruit development, with the lowest value in S3 senescent fruits (Figure 4). The signals that trigger ripening in fruits are not clear; the ethylene production rates follow a similar pattern with the respiration rates, which suggests that fruits might behave as non-climacteric. Non-climacteric fruits, such as grape and strawberry, do not exhibit a sharp peak in respiration, although a rise in ethylene is sometimes observed (Liu et al., 2015). Obviously, non-climacteric fruits have minimum capacity to synthesize ethylene, which may influence some physiological and molecular events during development and ripening of this class of fruit (Liu et al., 2015). By contrast, in climacteric fruits, such as tomato, banana, or apple, the onset of ripening is marked by an obvious respiratory burst linked to ethylene action, which is the primary cue mediating and controlling most aspects of climacteric fruit ripening at the physiological, biochemical, and molecular levels (Liu et al., 2015; Giovannoni et al., 2017).

**FIGURE 5:**
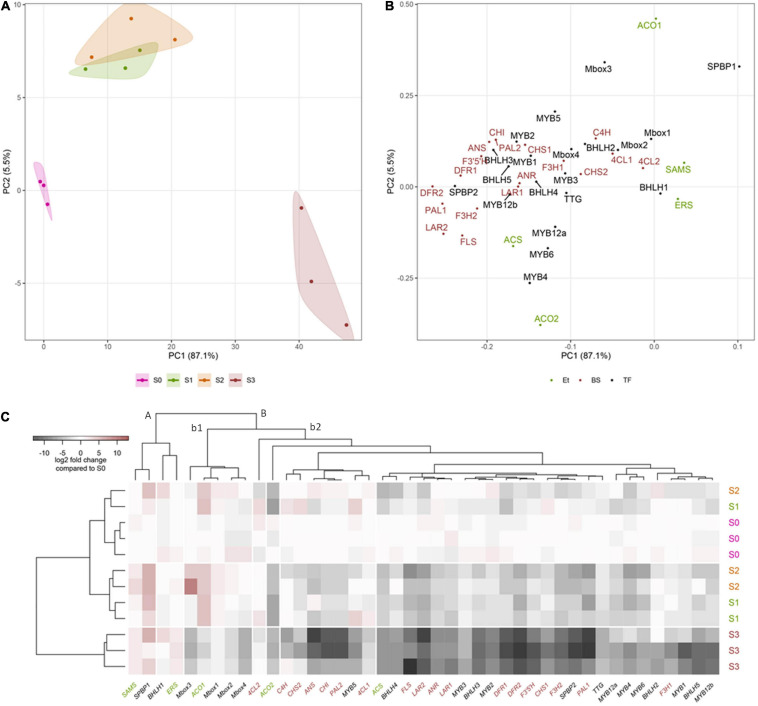
Chemometric analysis of the gene expression data for Cistus flavonoid-related biosynthetic genes (BS, red letters), transcription factors (TF, black letters), and genes involved in ethylene biosynthesis and sensing (Et, green letters). **(A)** Principal component analysis performed on log2 fold change expression data. The samples are colored by stage (S0–S3) as shown in the legend, and these groups are further encircled by a convex hull of the same color. **(B)** Principal component analysis variable loadings. **(C)** Heatmap of log2 fold change expression data. The samples (in rows) are arranged according to hierarchical cluster analysis (Ward’s method of cluster agglomeration) constructed using Euclidean distances (left tree), and the genes (in columns) are arranged according to hierarchical cluster analysis (cluster agglomeration using complete linkage) constructed using Pearson correlation distances (top tree).

Ethylene is synthesized from methionine (Figure 4) in a simple three-step biosynthetic pathway involving enzymes such as *S*-adenosyl methionine synthetase (SAMS), 1-amino cyclopropane-1-carboxylate synthase (ACS), and 1-amino cyclopropane-1-carboxylate oxidase (ACO). *S*-Adenosyl methionine, synthesized from methionine in a reaction catalyzed by SAMS, is converted by ACS into 1-amino-cyclopropane-1-carboxylic acid (ACC) that then is oxidized by ACO to ethylene (Liu et al., 2015). In this study, we utilized available transcriptomic information from *Cistus* to identify ethylene-related genes and known controllers of fruit development and ripening. Their expression in flowers and three stages of fruit development was investigated, aiming to better understand the maturation and ripening process in relation to flavonoid metabolism. We analyzed the expression patterns of genes involved in endogenous ethylene biosynthesis (*CcSAMS*, *CcACS*, *CcACO1*, and *CcACO2*) or in ethylene perception (ethylene response sensor, *CcERS*). The expression levels of *CcSAMS* were relatively stable during fruit development, with no significant changes from stages S0 to S3. Two ACO genes, *CcACO1* and *CcACO2*, displayed different expression profiles, in which the expression of *CcACO1* decreased in S3 stage, while the expression of *CcACO2* was relatively stable (Supplementary Figure 5). This negative correlation between the expressions of the two *CcACO* genes was also depicted in the correlation analysis of gene expression shown in Figure 5. On the other hand, the expression of *CcACS* gradually decreased during fruit development. In tomato fruit, the expression of *ACO* and *ACS* increased during tomato fruit ripening, which has been linked to ripening-associated ethylene production in climacteric fruits (Nakatsuka et al., 1998).

Ethylene induces a linear transduction pathway, which starts with the hormone perception by a specific ethylene receptor–ethylene response sensor, which then initiates a signaling cascade by releasing the block exerted by serine–threonine protein kinase constitutive triple response 1 (CTR1) on ethylene insensitive 2 (EIN2). This further activates a transcriptional cascade involving ethylene insensitive 3 (EIN3)/ethylene insensitive-like 1 (EIL1) as the primary TF and then ethylene response factors (ERFs) which, in turn, regulate genes underlying ripening-related traits, such as color, firmness, aroma, taste, and post-harvest shelf-life (Solano and Ecker, 1998; Solano et al., 1998; Ju et al., 2012; Cheng et al., 2013; Liu et al., 2015). In the absence of ethylene, CTR1 inhibits the activity of EIN2 (Kieber et al., 1993). *Cistus CcERS* expression slightly increased in S3 fruit (Supplementary Figure 5).

Transcription factors play critical roles in relaying ripening-inducing signals and controlling ethylene biosynthesis and signaling. TFs such as the SPBP, colorless non-ripening, ERF, as well as various MADS-box genes regulate downstream ripening genes (Vrebalov et al., 2002; Liu et al., 2015). In the TF networks that affect fruit ripening, MADS-box family TFs serve as the central regulators of ripening and are involved in early fruit development, maturation, and pre-ethylene ripening events in both climacteric and non-climacteric fruits (Yao et al., 1999; Boss et al., 2001; Tadiello et al., 2009; Seymour et al., 2011; Liu et al., 2015; Giovannoni et al., 2017). Within the present study, we traced the expression of four putative MADS-box genes identified in *Cistus* fruit transcriptome (*CcMbox1*, *CcMbox2*, *CcMbox3*, and *CcMbox4*). The expression of *CcMbox1* is significantly increased in S1 and S2 stages when compared to S0, and it decreased in S3 stage (Supplementary Figure 5). The expression of *CcMbox2*, *CcMbox3*, and *CcMbox4* is relatively stable during the stages S0–S2, and it significantly decreased in S3 stage (Supplementary Figure 5). This is in accordance with the proposed regulatory role of MADS-box TFs during maturation and pre-ethylene events in fruits.

### Chemometric Analysis of Gene Expression Data

A PCA combining gene expression studies revealed a clear separation between *Cistus* flowers and fruits (Figure 5A). PC1 and PC2 cumulatively explained 92.6% of total variance. Stages S0–S2 are clearly separated along PC1 (87.1% variability) from S3. Furthermore, flowers (S0) and S3 fruits are separated from S1 and S2 along PC2 (5.5%). In general, stages S1 and S2 are very similar in terms of gene expression profiles. The genes highly contributing to the diversification of *Cistus* samples along PC1 are *CcDFR2*, *CcPAL1*, *CcLAR2*, *CcSPBP2*, *CcFLS1*, *CcDFR1*, and *CcF3*′5′*H* (Figure 5B). On the other hand, *CcACO1*, *CcSPBP1*, *CcMbox3*, and *CcACO2* are the major variables contributing to the diversification of fruit developmental stages along PC2.

To analyze the transcriptional association among flowers (S0) and the three developmental stages of fruits (S1–S3), the data are also presented as a heat map of log2 fold changes (Figure 5C), arranged row-wise (samples) according to HCA constructed using Euclidean distances (Figure 5C, left) and column-wise (genes) according to HCA constructed using Pearson correlation distances (Figure 5C, top). Based on the presented tree (Figure 5C, left), samples from stage S3 form a separate homogenous cluster, while samples from stages S0–S2 are clustered together. As for the targeted gene expression linkages, ethylene-related biosynthetic genes (*CcERS* and *CcSAMS*) and TFs *CcBHLH1* and *CcSPBP1* form a separate cluster (A). The expression of these genes is stable or slightly increases during fruit ripening. All the other genes cluster together (B) and are separated into two sub-clusters (Figure 5C, top). The first sub-cluster (b1) comprises *CcACO1* and *CcMbox1* to *CcMbox4* TFs, while all the other genes belong to the second sub-cluster (b2). Within sub-cluster b2, two groups of genes could be diversified: (a) containing one EBG-*Cc4CL2* and (b) comprising all the other genes divided into two subgroups: (1) with *CcACO2* and (2) containing all the rest of the genes. Interestingly, the majority of EBGs (*Cc4CL1*, *CcCHI*, *CcPAL2*, *CcC4H*, and *CcCHS2*) cluster together with one LBG-*CcANS* and *CcMYB5* TF. On the other hand, the majority of LBGs (*CcF3H1*, *CcF3H2*, *CcFLS*, *CcLAR1*, *CcLAR2*, *CcANR*, *CcDFR1*, and *CcDFR2*) cluster close to two EBGs (*CcPAL1* and *CcCHS1*), five MYBs (*CcMYB1–4* and *CcMYB6*), *CcbHLH2* to *CcbHLH5*, *CcTTG*, and *CcMbox4* gene. The presented associations between genes indicate that EBGs are most likely regulated by *MYB4* to *MYB6*, while LBGs are under the control of *MYB1* to *MYB3* and corresponding bHLH TFs (*CcbHLH2* to *CcbHLH5*).

**FIGURE 6:**
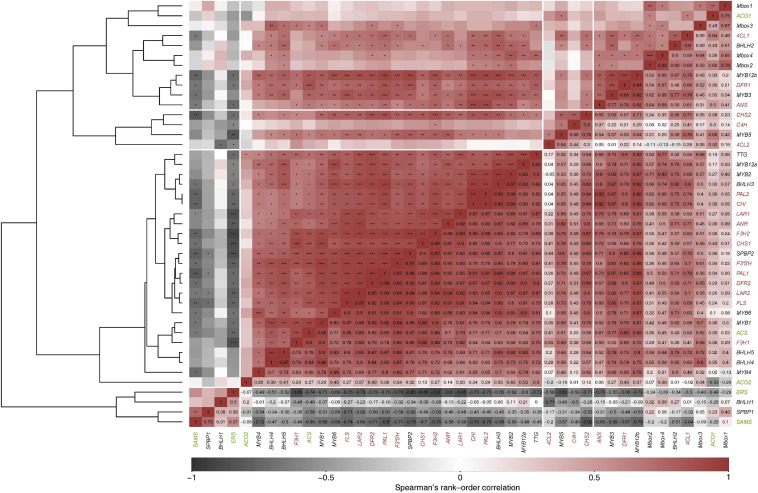
Correlation analysis of gene expression data. The correlation matrix was constructed using Spearman’s rank-order correlation. Spearman’s correlation coefficients are shown in the lower triangle, while the significance of the association samples using a two-sided test (*p* < 0.05) is denoted by asterisks in the upper triangle. The genes are ordered according to hierarchical cluster analysis (cluster agglomeration using complete linkage) constructed based on 1-corsp dissimilarity matrix (left tree).

A pairwise correlation analysis of the expression patterns between flavonoid biosynthetic genes and TFs was carried out to identify TFs that are co-expressed with the biosynthetic genes (Figure 6). Positive correlation has been observed between all the analyzed biosynthetic genes (EBGs and LBGs) and TFs (MYBs, bHLHs, and TTG), indicating the transcriptional regulation of flavonoid biosynthesis in *Cistus* fruit. The exception is the expression of *CcbHLH1*, which was negatively correlated with the analyzed biosynthetic genes, especially with *Cc4CL2* and *CcCH4*. The positive correlation in the expression levels between MYB, bHLH, and TTG TFs indicated the role of MBW activation complex in the regulation of PA synthesis in fruits. MBW complexes might be involved in the developmental regulation of the flavonoid pathway at the transcriptional level, especially in the LBGs (DFR, ANR, ANS, LAR, F3H, F3′5′H, and FLS). EBGs (PAL, C4H, 4CL, CHS, and CHI) are probably less affected by the activities of these complexes. The exception again seems to be *CcbHLH1*, the expression of which was negatively correlated with the expression of other MYB, bHLH, and TTG TFs. Thus, it appears that PA biosynthesis in *Cistus* fruits is controlled by a network of MYBs (activators and repressors) and bHLHs, which could allow for a more subtle regulation of the transcriptional mechanism through different combinations of TFs within the MBW complex.

Among the analyzed ethylene-related genes, *CcACO1*, *CcACS*, and, to some extent *CcACO2* were positively correlated with most of the flavonoid biosynthetic genes and TFs, with the exception of *CcSPBP1* and *CcbHLH1* (Figure 6). On the other hand, *CcERS* and *CcSAMS* were significantly negatively correlated with all the biosynthetic genes and majority of TFs but were positively correlated with *CcSPBP1* and *CcbHLH1*. The only significant positive correlation of *CcSAMS* was with *CcSPBP1*.

### Correlation Analysis Between Metabolomics and Gene Expression Data

**FIGURE 7:**
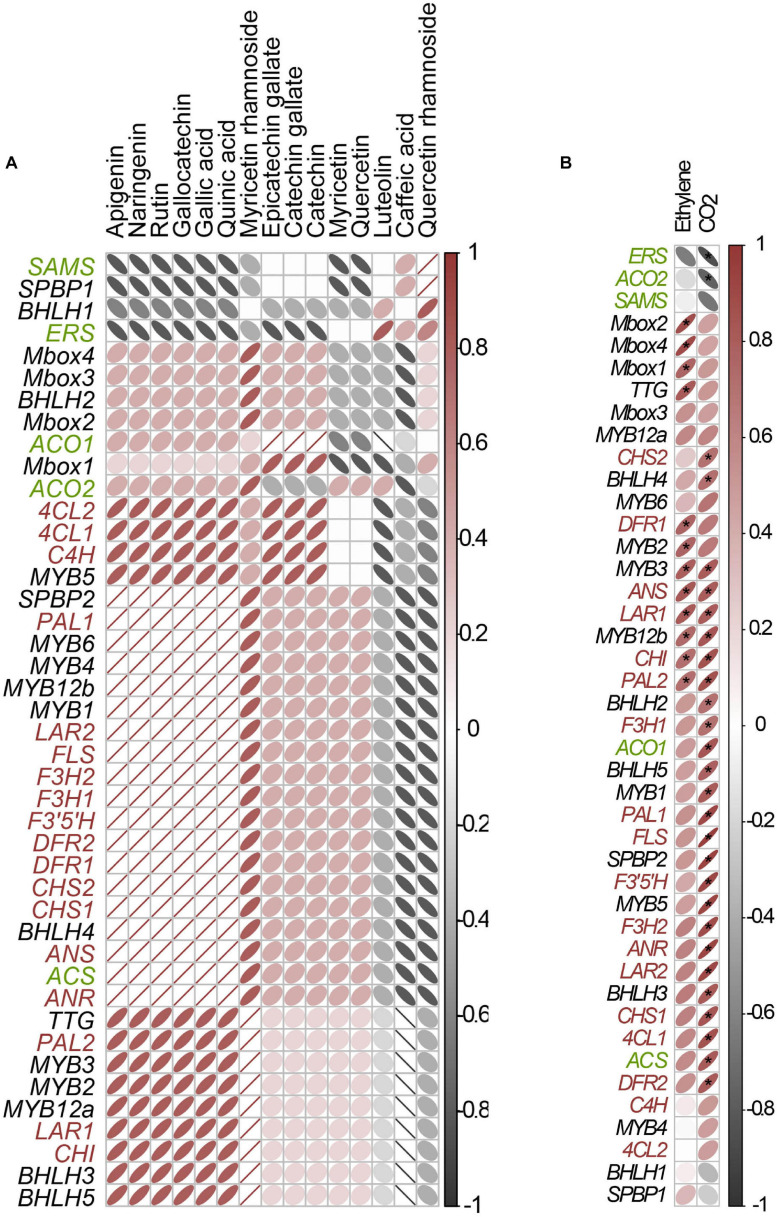
**(A)** Pairwise correlation analysis of gene expression and phenolics quantitative data in flowers (S0) and fruits of three developmental stages (S0–S3). Since different biological samples were used for metabolomics and expression measurements, the correlation was performed on averaged data by developmental stage. **(B)** Pairwise correlation analysis of gene expression and ethylene production and respiration (CO_2_) data in *Cistus* fruits of three developmental stages (S1–S3). For both **(A,B)**, the correlation matrix was constructed using Spearman (rank) correlations between compound quantity and relative gene expression. Asterisks denote significant (*p* < 0.05) association between paired samples using a two-sided test.

The expression of structural genes is coordinately regulated and well correlated with metabolite pools, supporting the hypothesis that the biosynthesis of flavonoids during *Cistus* fruit development is controlled at the transcriptional level (Figure 7A). All the structural gene transcripts were more abundant in flowers (S0) and during the first stages of fruit development (S1 and S2) when the majority of targeted flavonoids accumulated and the transcripts of the TFs were at a high level.

The exceptions are correlations between caffeic acid (14), quercetin 3-*O*-rhamnoside (43), and luteolin (50) content on one side and analyzed gene expressions on the other (Figure 7A). Of the five bHLH candidates analyzed, *CcbHLH1* was the only one positively correlated with the amount of 14 and 50, while both *CcbHLH1* and *CcbHLH2* were positively correlated with 43. The above-mentioned compounds were also positively correlated with *CcSPBP1* and with some ethylene-related genes (*CcERS* and *CcSAMS*). However, such correlations should be considered with caution, especially in complex metabolic pathways in which the accumulation of the intermediates is balanced between the rate of biosynthesis and further utilization and conversion to other molecules. For example, the amount of naringenin (52) resulted from the activity of biosynthetic genes upstream of the pathway (PAL, 4CL, C4H, and CHI) but is also influenced by the rate of its conversion into other flavonoids as mediated by the activity of downstream enzymes (Figure 1). As stated before, 52 is considered the branching point for the biosynthesis of different flavonoid classes. In the same way, the detected concentrations of other intermediates of the pathway, including several detected aglyca, should be considered.

A correlation analysis of flavonoid-related biosynthetic genes, ethylene-biosynthetic genes, and TF expression patterns on the one hand and ethylene production/respiration on the other was carried out to identify possible associations between flavonoid and ethylene metabolism (Figure 7B). Ethylene production rate and respiration were positively correlated with the expressions of *CcACS* and *CcACO1* and negatively correlated with *CcACO2*, *CcERS*, and *CcSAMS* expressions. On the other hand, the expression of the majority of flavonoid biosynthetic genes and TFs analyzed was positively correlated with the level of ethylene production and respiration (Figure 7B). Significant positive correlations were observed for endogenous ethylene production and *CcDFR1*, *CcANS*, *CcLAR1*, *CcCHI*, and *CcPAL2* transcript levels in *Cistus* fruits. In strawberry, *FabHLH98* is responsive to abiotic stress with the implementation of ABA and ethephon, which, according to Zhao et al. (2018), suggests its involvement in fruit ripening. Ethylene has strong effects on persimmon fruit ripening (Nakano et al., 2003) and on the removal of astringency resulting from high PA contents (Yin et al., 2012). ERFs induce a decrease of PAs in persimmon *via* the ethylene pathway (Min et al., 2012). As previously reported, ethylene signaling is involved in the regulation of *VvCHS*, *VvF3H*, *VvDFR*, *VvLDOX*, and *VvUFGT* expression in grape (El-Kereamy et al., 2003). The transcription of *VvCHI1*, *VvCHS3*, *VvDFR*, *VvLDOX*, *VvUFGT*, and *VvMYBA1* was up-regulated by ethephon, a commercial growth regulator that is quickly converted to ethylene, which resulted in the activation of PA and anthocyanin biosynthesis in grape berries (Liu M. Y. et al., 2016). Since ethylene production and respiration are positively correlated with the expression of the majority of flavonoid biosynthetic genes and related TFs, there are indications that ethylene biosynthesis and signaling might be involved in the biosynthesis of flavonoids in *Cistus* fruits. Further studies are needed in order to find connections between these two processes.

### Isolation and Heterologous Expression of Flavonoid Hydroxylase (F3′5′H) in Yeast

**FIGURE 8:**
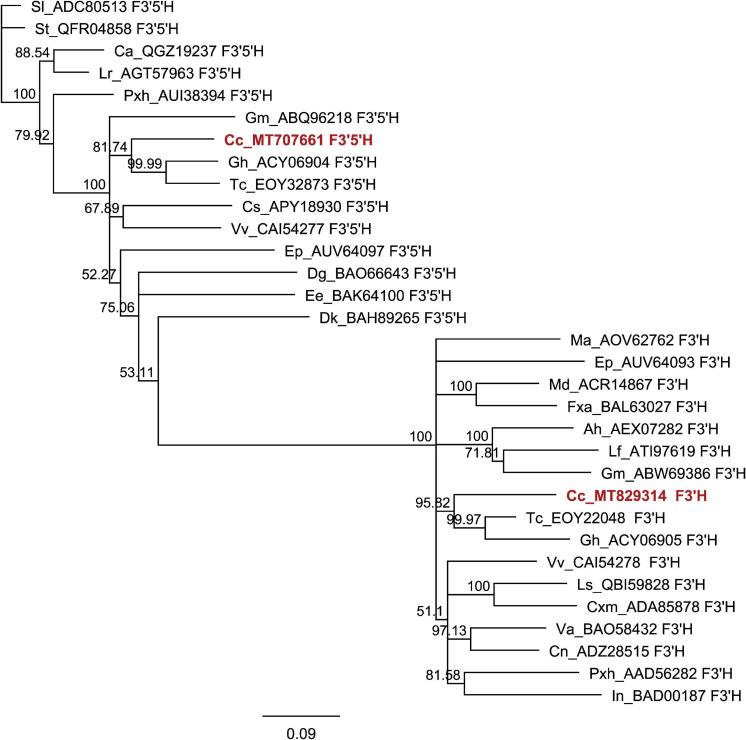
Protein sequences for F3′H and F3′5′H in various plant species available in GenBank were selected, including those of *Cistus creticus*. Sequences were aligned using ClustalW and phylogenetic distance tree analysis followed in Geneious Tree Builder, with Jukes–Cantor genetic distance model and neighbor-joining method. A consensus tree was built using bootstrap resampling tree method, with 10,000 replicates and majority greedy clustering. The scale bar indicates 0.09 amino acid substitutions per site. Bootstrap values are shown in nodes. The accession numbers displayed are given in Supplementary Table 3.

**FIGURE 9:**
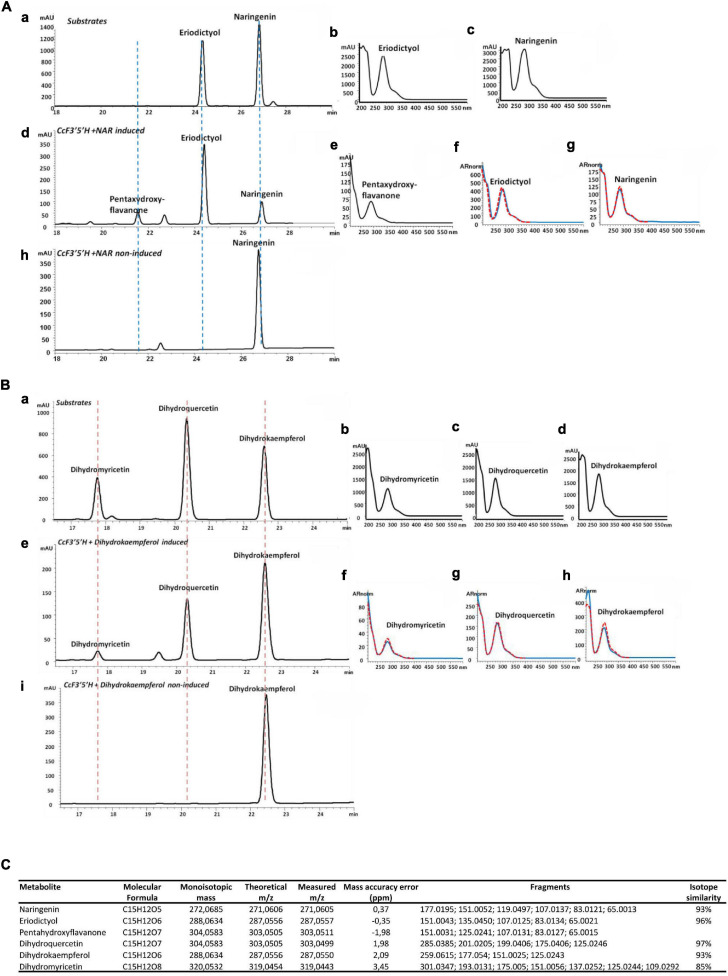
Functional characterization of *Cistus creticus F3*′5′*H* gene. **(A)** Detection of products in methanolic extracts of yeast culture feeding assay with naringenin after biotransformation with F3′5′H gene. Ultra-performance liquid chromatography (UPLC)–photodiode array (PDA) chromatograms of (a) the substrate naringenin and the product eriodictyol and their corresponding absorption (b) and (c); (d) yeast culture transformed with CcF3′5′H flavonoid hydroxylase after 72 h of feeding with 5 μM naringenin and the alignment of the identified absorption of the products pentahydroxyflavanone (e) and eriodictyol (f) and the substrate naringenin (g) (line in blue) with the external standards from the diode array detector (DAD) library (line in red); (h) negative control yeast culture transformed with CcF3′5′H after 72 h of feeding with 5 μM naringenin without induction with galactose. **(B)** Detection of products in methanolic extracts of yeast culture feeding assay with dihydrokaempferol after biotransformation with F3′5′H gene. UPLC–PDA chromatograms of (a) the substrate dihydrokaempferol and the products dihydroquercetin and dihydromyricetin and their corresponding absorption (b), (c), and (d); (e) yeast culture transformed with CcF3′5′H flavonoid hydroxylase after 72 h of feeding with 5 μM dihydrokaempferol and the alignment of the identified absorption of the products dihydroquercetin and dihydromyricetin and the substrate dihydrokaempferol (h) (line in blue) with the external standards dihydroquercetin, dihydromyricetin, and dihydrokaempferol from the DAD library (line in red); (i) negative control yeast culture transformed with CcF3′5′H after 72 h of feeding with 5 μM dihydrokaempferol without induction with galactose. **(C)** Table presenting the relevant data of HDMS QTOF MS identification of all six metabolites.

Hydroxylation appears as one of the crucial steps defining the final metabolite pattern in *Cistus* flowers and fruits. Besides 3′,4′-hydroxylated flavonols and F3Os, such as quercetin, catechin, and epicatechin derivatives including the respective PAs (i.e., procyanidins), 3′,4′,5′-hydroxlated counterparts (myricetin, gallocatechin, epigallocatechin, and its derivatives and related prodelphinidins) were found as major metabolites in the investigated tissues. These findings indicate the significant involvement of active hydroxylases responsible for the decoration of the basic flavonoid structure. To identify putative genes coding for functional F3*′*5*′*H proteins from *C. creticus*, a blast similarity search was performed. Functionally characterized genes from 42 plant species were selected and retrieved from NCBI public database (Supplementary Table 3). The local tblastn against RNA-seq from *C. creticus* fruit revealed two sequences for putative flavonoid hydroxylases, named c15585 and c24086, respectively. To assign a putative function to these genes, the retrieved candidate genes were used to generate a phylogenetic tree (Figure 8). The tree demonstrated that the putative contig c15585 is more closely related to F3′5′H genes from *Theobroma cacao* and *Gossypium hirsutum* and was clustering within the group of functionally characterized F3′5′Hs from various plant species. The candidate c24087 grouped clearly within the F3′H genes, branching again close to the same species (*T. cacao* and *G. hirsutum*). However, according to Seitz et al. (2006), a prediction of the *in vivo* function of flavonoid hydroxylases from phylogenetic sequence analysis could be, at least for candidates from Asteraceae family, misleading, and an enzymatic characterization of the encoded protein is suggested. Therefore, heterologous expression of the putative flavonoid 3′,5′-hydroxylase in yeast and *in vivo* bioconversion of selected substrates were performed to determine the exact function of this important step in determining the substitution pattern of major *Cistus* metabolites. The yeast strain INVSc1 was used to transform and over-express the candidate c15585, cloned into the expression vector pYES2. The induced transformed yeast cells were fed with two potential substrates, naringenin and dihydrokaempferol, dissolved in DMSO (Figure 1). The UPLC–PDA analysis of yeast culture extracts after 24, 48, and 72 h of incubation revealed that the expression of c15585 gene produced a functional enzyme with the predicted activity, validated by the presence of the respective 3′,4′,5′-hydroxylated products pentahydroxyflavanone and dihydromyricetin (Figure 9). Due to a lack of authentic standard, pentahydroxyflavanone was tentatively identified by its retention time behavior and UV profile, which is characteristic for flavanones. In all extracts, the first expected reaction products, eriodyctiol and dihydroquercetin, were also detected. Furthermore, the identity of all six metabolites including pentahydroxyflavanone was confirmed by means of MS analysis using UPLC coupled *via* an ESI interface to a Synapt HDMS QTOF MS (Figure 9–C and Supplementary Figure 6). The mass accuracy error was, for all compounds, below 5 ppm. Compared with the respective standard, as available, the isotope similarity was above 70%, which allows the unambiguous identification of the given compounds (Figure 9–C). Detailed fragmentations of the compounds at two different energy levels are given in Supplementary Figure 6. These results clearly identify c15585, deposited in GenBank with number MT707661, as a functional F3′5′H involved in the specific hydroxylation of flavanones and/or dihydroflavonols, determining the final hydroxylation pattern of flavonols, F3Os, and PAs and most probably also of anthocyanins, which were not analyzed in this study.

Furthermore, the identified activity is in line with the phylogenetic clustering of the putative flavonoid hydroxylases’ protein sequences. Therefore, we can postulate that the F3′5′H enzyme encoded by c15585 is involved in converting the metabolic flux toward biosynthesis of myricetin and its derivatives and through the delphinidin branch within the flavonoid biosynthesis network in *C. creticus*.

## Conclusion

Being a rich source of flavonols, F3Os, and PAs, *C. creticus* subsp. *creticus* fruit represents a convenient model system to study the metabolism of these compounds in relation to development and ripening processes. Patterns of changes in the flavonoid content and expression profiles of biosynthetic genes and TFs during *Cistus* fruit development suggest a coordinated regulation of gene expression at the transcriptional level. The results propose a significant role of flavonoid hydroxylases in determining the content and ratio of major flavonoid compounds belonging to the groups of flavanones, flavones, dihydroflavonols, and flavonols and indirectly of F3Os and PAs. The function of the most important gene belonging to this group, *CcF3*′5′*H*, which is responsible for conducting the metabolic flux through delphinidin branches of the biosynthetic pathway, is confirmed. The involvement of a network of MYBs (activators and repressors) and bHLHs in the subtle regulation of the transcriptional mechanism through different combinations of TFs within the MBW complex is proposed. Although the results suggest the involvement of ethylene biosynthesis and signaling in the biosynthesis of flavonoids in non-climacteric *Cistus* fruit, further studies are needed in order to confirm the connections between these two processes.

## Data Availability Statement

The Data has been deposited to GenBank: GenBank MT707661.

## Author Contributions

NA, AP, AntK, SM, DM, and AngK conceived and designed the experiments. NA, EP, AP, AntK, CV, PA, MS, AthK, SK, ES, and SM performed the experiments. UG and DM performed the phytochemical characterization of samples. MD was responsible for the statistical data analysis. DM, NA, ES, SM, and AngK organized and wrote the manuscript with editing from all the authors.

## Conflict of Interest

The authors declare that the research was conducted in the absence of any commercial or financial relationships that could be construed as a potential conflict of interest.

## Footnotes

1 https://www.ncbi.nlm.nih.gov/tools/primer-blast/
