# Supplementary figures and images for: Comparative Metabolite and Gene Expression Analyses in Combination With Gene Characterization Revealed the Patterns of Flavonoid Accumulation During Cistus creticus subsp. creticus Fruit Development
Source: Front Plant Sci. 2021 Mar 26;12:619634. doi: 10.3389/fpls.2021.619634 (PMC8034662; doi:10.3389/fpls.2021.619634)

compound quantity [ $\mu\text{g} / 100 \text{ mg DW}$ ]

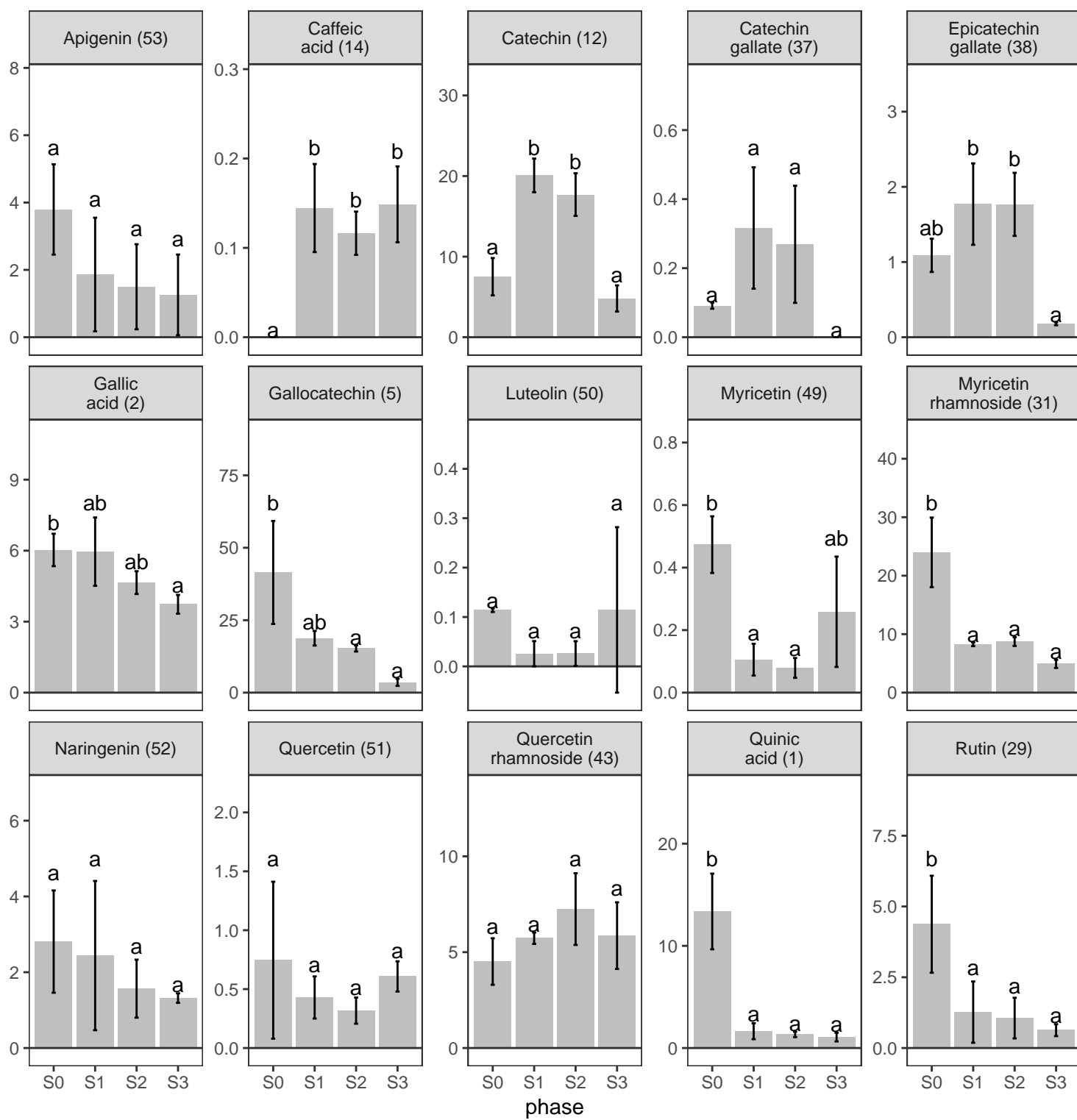

Supplement: Supplementary file 4 [file Data_Sheet_1.PDF]

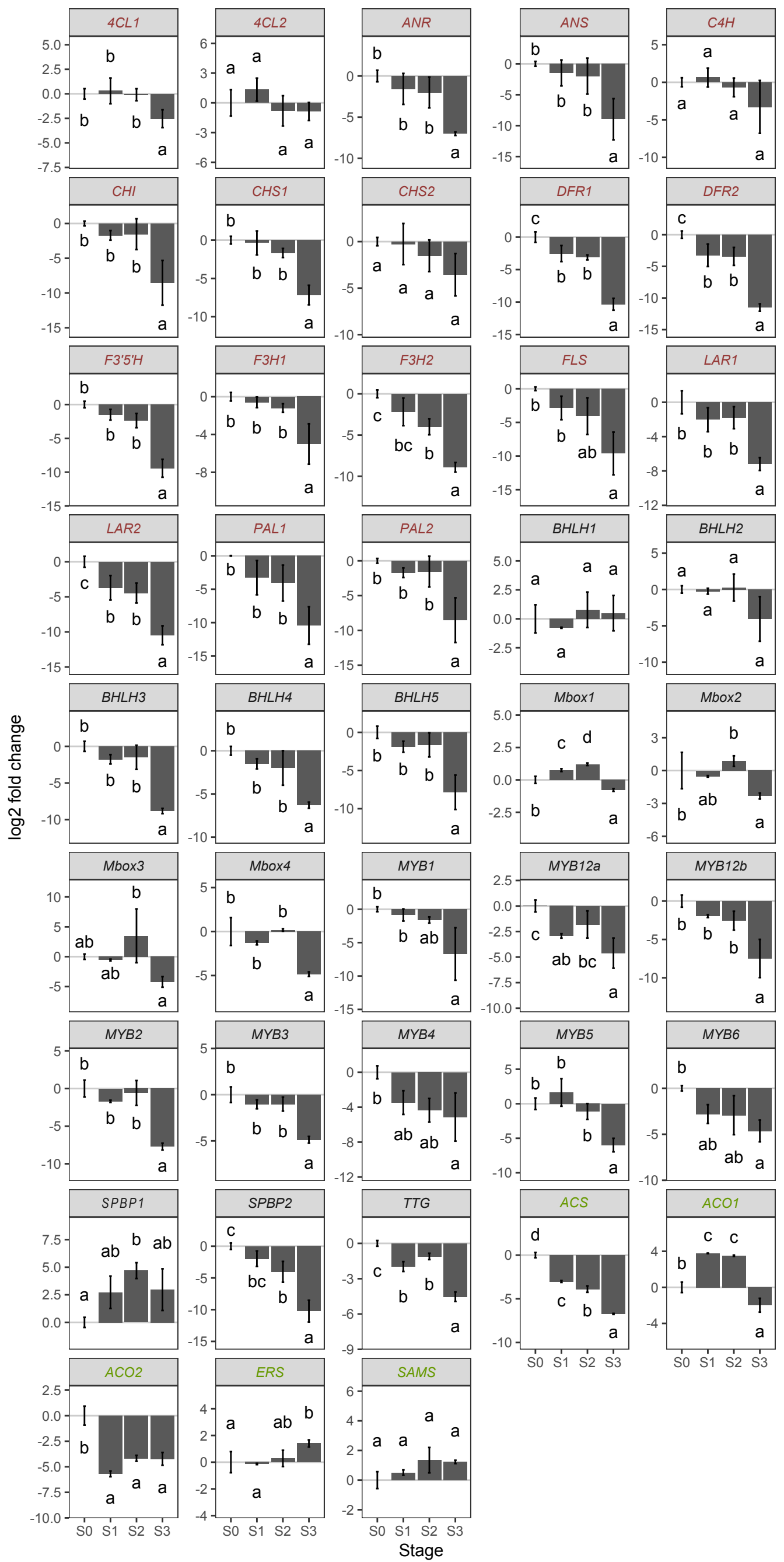

Supplement: Supplementary file 5 [file Data_Sheet_2.PDF]
