# Supplementary material for: Comparative Metabolite and Gene Expression Analyses in Combination With Gene Characterization Revealed the Patterns of Flavonoid Accumulation During Cistus creticus subsp. creticus Fruit Development
Source: Front Plant Sci. 2021 Mar 26;12:619634. doi: 10.3389/fpls.2021.619634 (PMC8034662; doi:10.3389/fpls.2021.619634)
Supplement: Supplementary file 6 [file Data_Sheet_3.PDF]

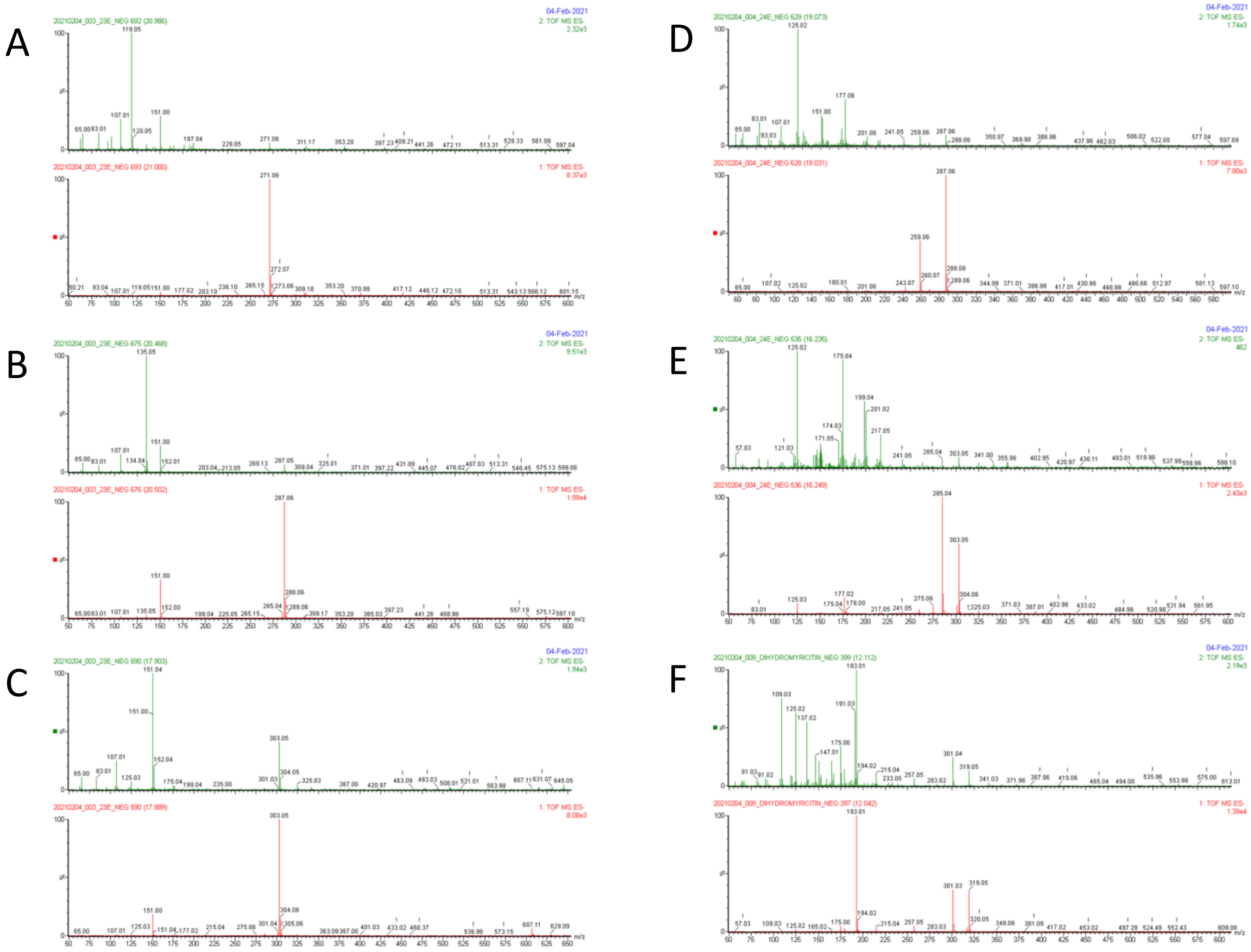

Supplementary Figure S6. Fragmentation of the six key compounds of enzymatic studies of Cistus F3'5'H recombinant protein at two different collision and trap collision energies: upper spectra (green lines)- high energy with 6 V for collision and 30 V for trap collision; lower spectra (red lines)- low energy with 4 V for collision and 6 V for trap collision. A - Naringenin; B - Eriodictyol; C - Pentahydroxyflavanone; D- Dihydrokaempferol; E - Dihydroquercetin: F - Dihydromyricetin;
