# Supplementary material for: Comparative Metabolite and Gene Expression Analyses in Combination With Gene Characterization Revealed the Patterns of Flavonoid Accumulation During Cistus creticus subsp. creticus Fruit Development
Source: Front Plant Sci. 2021 Mar 26;12:619634. doi: 10.3389/fpls.2021.619634 (PMC8034662; doi:10.3389/fpls.2021.619634)
Supplement: Supplementary file 7 [file Table_1.docx]

Table 1. UHPLC/DAD/(−)HESI-MS^2^ data of targeted compounds in methanol extracts of *Cistus creticus* spp. *creticus* flowers and fruits. Relative intensities of the main diagnostic MS^2^ fragments are presented.

|  | | -HESI-MS | | | DAD data | |
| --- | --- | --- | --- | --- | --- | --- |
| Peak | Assignment | Rt (min) | [M-H]^-^ [*m/z*] | MS^2^ fragments [*m/z* (relative intensity in %)] | Rt (min) | λ_max_ (nm) |
| **2** | **Gallic acid^S,R^** | 0.70 | 169 | 125 (10), 79 (100) | 0.47 | 260 |
| **1** | **Quinic acid ^S,R^** | 0.48 | 191 | 191 (40), 171 (<5) | 0.60 | 270 |
| **5** | **Gallocatechin^S,R^** | 1.18 | 305 | 221 (45), 178 (20) | 1.07 | 230, 280 |
| **12** | **Catechin^S,R^** | 2.29 | 334* | 289 (100), 97 (30) | 2.23 | 230, 280 |
| **14** | **Caffeic acid^S,R^** | 2.60 | 179 | 134 (85), 117 (10) | 2.58 | 230, 330 |
| **31** | **Myricetin rhamnoside ^R^** | 3.94 | 463 | 316 (100), 300 (60) | 3.89 | 260, 360 |
| **29** | **Quercetin rutinoside (Rutin)^S,R^** | 3.88 | 609 | 301 (20), 300 (100) | 3.82 | 260, 350 |
| **37** | **Catechin gallate^S,R^** | 4.03 | 441 | 303 (5), 289 (100) | 3.97 | 230, 280, 370 |
| **38** | **Epicatechin gallate^S,R^** | 4.08 | 441 | 303 (5), 289 (100) | 4.02 | 230, 280, 370 |
| **41** | **Quercetin rhamnoside (Quercitrin)^S,R^** | 4.44 | 447 | 284 (100), 255 (65) | 4.37 | 270, 340 |
| **46** | **Myricetin^S,R^** | 4.80 | 317 | 151 (100), 109 (20) | 4.72 | 260, 360 |
| **47** | **Luteolin^S,R^** | 5.31 | 285 | 151 (15), 133 (100) | 5.30 | 260, 350 |
| **48** | **Quercetin^S,R^** | 5.38 | 301 | 179 (<5), 151 (100) | 5.41 | 260, 360 |
| **50** | **Apigenin^S,R^** | 5.80 | 269 | 149 (10), 117 (100) | 5.72 | 260, 340 |
| **49** | **Naringenin^S,R^** | 5.83 | 271 | 151 (15), 119 (100) | 5.75 | 260, 340 |

Masses used in SRM (Selected Reaction Monitoring) experiments for the quantification of compounds are presented in bold.

Abbreviations: S-confirmed by standard; R-confirmed by references.

*Pseudomolcular ion of catechin [M-H+formic acid]^-^ [*m/z*]
