# Supplementary material for: Comparative Metabolite and Gene Expression Analyses in Combination With Gene Characterization Revealed the Patterns of Flavonoid Accumulation During Cistus creticus subsp. creticus Fruit Development
Source: Front Plant Sci. 2021 Mar 26;12:619634. doi: 10.3389/fpls.2021.619634 (PMC8034662; doi:10.3389/fpls.2021.619634)
Supplement: Supplementary file 8 [file Table_2.docx]

**Supplementary Table 2. Primer sequences and combinations used in qPCR analysis.**

| Target gene | Primer sequence (5’-3’)  Forward | Primer sequence (5’-3’)  Reverse | Gene type |
| --- | --- | --- | --- |
| ELF | ACGATCCTGCCAAGGGAGCAGCC | CGTCCCAATGGAGGGTACTCGGAGA | Reference gene |
| PAL1 | AGGGAAATGGCACAAAATCCCCGGGA | AATGGCGGCGGCAACAACGCTCTTA | Flavonoid-related biosynthetic genes |
| PAL2 | CATCACGAGAACGGGTCGTTATGTGTGAGTC | GACAGCTCCACCGTGACATCCAATTCACTAG |  |
| C4H | ACGCCGTGTTGGGAGTAGGCCA | GCCAGCCACCAAGCGTTGACCA |  |
| 4CL1 | CTCCCGAATTGTCCGGAATTCGTGCTGGC | CGGTAATCAGTTTCACGTCGTTTTCCGTTGCG |  |
| 4CL2 | CGCTTTCCCCAATAAACCGGCGCTG | GGCGATTTTGTCGGCCAATTCGGGGACG |  |
| CHS1 | GTTCAAGCCTTTAGGCATCTCAGATTGGAACTCAC | CTCCTGTTGTCTCGAGCCCATCTTCTTTTGACTTCC |  |
| CHS2 | GTTTTCGCCGATCGGAATCAACGACTGG | CCGGTGGTTCTTTTCCCTTCCTCCAGCGAT |  |
| CHI | TGCTGTAGGGACAGATTGGCTGCT | TGCTGAGAGGTTGTTGGCCAGGGA |  |
| F3H1 | GAACTCGAGCAGGTTGTCAATTGCAACGTTC | GGATTTGTTCCAGAGGCTTGGCATGAGG |  |
| F3H2 | CAATAGCAGGTTATCCATAGCTACATTCCAGAACCC | CTCAATTGGCTTAGGATCCAACTTAGCCTTCTCC |  |
| F3’5’H | TGCCGCAGCCGCCCTCTTTT | GGCGGCATCCGGTGTGGAGG |  |
| DFR1 | GCATGTCTCACATATACCTCTTCGAGCACCC | CTTCTTTTCCCTGCAAGTTTCCACCGCC |  |
| DFR2 | TCCTTTCCTCATGTCCTCAATGCCTCCAAGC | TCGGGGTATTTCTTCTTAAGCATTCTCGAGATATCG |  |
| FLS | ACGAGGTTCCGGGCCTCCAAGTT | ACCATCAACTCCCCTGGCGGCT |  |
| LAR1 | ATTCTCCTCCGTCCCACCACCGCTCTCAA | GGAAGTTGGTCCAATAACGTTCCCCCACC |  |
| LAR2 | CCGTTTCCGAGGATGACCTTCTCCAAAGAG | CATGCCGTTCATGGTGACTGCCGGCTT |  |
| ANS | GCGGCCACAGATTGGGGCGT | CCCACTCGAGCTGCCCGCTAGT |  |
| ANR | CCGGACTGCCTTTTTCCCGCTATGC | CCAGAGCATCGCGTGGGGTTTTCC |  |
| ACO1 | GGGCCTTAGGGCACATACTGATGCAGGA | GGGCATTTTTATCAGTCTCAAGCAAAGTTGGTGCTG | Ethylene biosynthetic genes |
| ACO2 | ATCAAAGGGCTGCGTGCCCACAC | CAATGGACATCCTTCCGGAGCCTTGAGTC |  |
| ACCS | AGCCACAAGGCGCAAGGTCTTCACT | TCCGAGCAGTGGAAAGAGGAGCCG |  |
| SAMS | CGCCATTGGTGTCCCCGAGCC | GGGGCTTCACGACCTCCCAGGTG |  |
| ERS | GCGCCTTGTGGATGCCAACTCGT | ACGCACAGCGACCACCTCTCCT |  |
| Mbox1 | GAAAACAGCGTCGAGCGCGAAACACAG | CAGAGTCCTTGAACTGGTTTAAAGCTTTGTCCTTC | Transcription factors |
| Mbox2 | GGGAGAAACCAGCTCCAAGTATCATGGGATGG | GACTTGTTGTTGACCATGGGAATGGGATGGAC |  |
| Mbox3 | ATTCTCCACAGAGGAAATCTGCTGGGAGGG | CCATTGTTGGAGGTGTCTGCGCAAGAC |  |
| Mbox4 | GCTGGAAGCATCCTTGAAGCAGATAAGGTCTAC | GCTGCCGTCCCATGTTAGTTGAAGAGGA |  |
| MYB1 | GCAGGGAGATTGCCTGGACGCAC | TGGCACCACCCGTGGAATTCGGA |  |
| MYB2 | GCTGCAGGGCTCCATCGTTGCG | AGGGCGGAGGGTCACCAAACGG |  |
| MYB3 | CCCCCAGACTCACCGCCCCC | TTCTGCTCCCGCCTCCGCCC |  |
| MYB4 | TTGCGGGGAGGCTACCGGGG | TGGGCAAGTGGCTTGGTCCGGT |  |
| MYB5 | GGTCCCTACTTCGACACCATCTACATCATCATC | CAAAAGGAGAGCCCATACGTCCAATAATCTTGATC |  |
| MYB6 | GCTCCTCCTGCTTCCCCGGCT | TGGCATCGAGGCTCCGGCCAA |  |
| BHLH1 | TGCAGCAGTCTGTGAACGGCTTGAG | TGTGTCGGGCTTGGGAGGACGA |  |
| BHLH2 | TGACGAACGCAGTCATGGCGCT | TGTTCTGTGGCACCCCGGGACT |  |
| BHLH3 | CCGCCGCCGGGAGAAGCTG | TGCGGCCCGACCACGATCCG |  |
| BHLH4 | CCCCGCCACCTCCTCCGACC | GCCGCCGCGGATTGAGCGTT |  |
| BHLH5 | GCCGCGCTGGCTGGTCAGTCT | TGGCAGCATCGCAGAGCAACCG |  |
| MYB12_1 | ATCCGCGACGACAAAGACAACGCC | ACCACGCTTAACGGATGGCCGGA |  |
| MYB12_2 | CAGCGGAGAAGCCCGGCCC | ACCCTTCTCCGTCGTCATCAACGCC |  |
| SPBP1 | GCGTCGTCGCCGCCTTCTTGT | GCCTGCGACAGCTCCTCTTCGC |  |
| SPBP2 | TCCGGTGGATCTACGGCGCCAC | TCCGGCCTTTCGTCTCCGGCA |  |
| TTG | GGCCAACGCATCGCTCTGGGG | ATTCCCGAACCTCCCAAAGGCGGA |  |
